# Hypopyon from observation to insight: a multidimensional system involving pathogenesis, lesion nature, clinical manifestations and diagnosis and treatment

**DOI:** 10.3389/fmed.2026.1935253

**Published:** 2026-10-30

**Authors:** Ruoqing Li, Lijun Qu, Shiyang Niu

**Affiliations:** 1 The Second Affiliated Hospital of Harbin Medical University, Harbin, China; 2 Department of Ophthalmology, The Second Affiliated Hospital of Harbin Medical University, Harbin, China

**Keywords:** classification, diagnosis, endophthalmitis, hypopyon, infection, keratitis, pathogenesis

## Abstract

Hypopyon is a pivotal clinical sign guiding diagnosis, treatment selection and efficacy evaluation in ophthalmology. However, its overlapping etiologies, atypical presentations of rare emerging pathogens, and the lack of rapid differential tools between infectious and sterile inflammation frequently lead to delayed diagnosis, inappropriate management and irreversible visual impairment. Recent advances in multiplex molecular pathogen detection, quantitative AS-OCT imaging, and mNGS offer new perspectives for investigating the etiology of hypopyon. However, evidence that these modalities refine multi-dimensional phenotypic stratification remains limited, and challenges including variable diagnostic accuracy and the absence of standardized interpretation protocols persist. This review summarizes the pathogenesis, clinical features and classification framework of hypopyon, covering infectious/non-infectious origins, primary/secondary occurrence, and true hypopyon/pseudohypopyon. It provides an up-to-date, evidence-based reference for accurate differential diagnosis and individualized clinical management.

## 1 Introduction

The English term “hypopyon” combines the Greek phrases hypo-, meaning under, below and pyon, meaning pus. Thus, its literal translation is pus settled below. The term's etymology captures the essential clinical feature most accurately—a layered accumulation of purulent material in the inferior aspect of the anterior chamber. So, this defines the term for the ocular sign. The Collins English Dictionary records that the term “hypopyon” is of Greek origin and entered the English medical lexicon as early as 1,650–1,660, with its meaning referring to the intraocular accumulation of pus or leukocytes. In 1930, a Greek doctor Adamantiades first described cases of “recurrent pus in the anterior chamber with oral ulcers” and linked recurrent hypopyon to systemic disease (1). The 20th Century saw a further understanding with the slit-lamp biomicroscope in common use. This tool helped the clinicians see certain subtle features of this hypopyon, which could help lay the technical grounds for phenotype classification down the line. With the turn of the century, higher-end molecular diagnostics like PCR and imaging like OCT and gonioscopy have driven the terms into a phase of fine-tuning and standardization. In 2005, Professor Evereklioglu portrayed the entity of “pseudohypopyon” and clinically differentiated it from classic inflammatory hypopyon (2). By the year 2023, “naming-meshing” was further promoted to specific characteristics of hypopyon, which include visibility, mobility and color with underlying causes. The development of the term “hypopyon” reveals its progression from a mere adjective to a fully-fledged medical term with anatomical location material nature, pathological mechanism and diagnostic import (3).

## 2 Method of literature search

This work is structured as a narrative review focused on the pathogenesis, clinical features, and classification system of hypopyon. The literature search was performed in the PubMed database. The primary search keyword was hypopyon, and supplementary targeted searches were conducted for related clinical entities including endophthalmitis, anterior uveitis, and toxic anterior segment syndrome, to identify all relevant studies published between 1979 and 2026. Studies were selected based on clinical relevance, methodological rigor, and timeliness. Priority was given to authoritative peer-reviewed reviews, large prospective and retrospective cohort studies, international clinical practice guidelines, and high-quality original research articles. Classic landmark studies that established core concepts in the field were also included to provide a complete historical perspective of the field's evolution. Articles that were tangentially related to the topic, contained redundant findings, or had significant methodological limitations were excluded. After screening, a total of 140 core references were included in this review. The purpose of this narrative review is to systematically integrate current evidence on the etiology, pathogenesis, differential diagnosis, and classification of hypopyon, and to provide an evidence-based framework to guide clinical decision-making. It is not intended as a systematic review or meta-analysis with quantitative pooling of outcomes.

## 3 Etiological classification: infectious vs. non-infectious

### 3.1 Infectious hypopyon

Direct penetration by infective pathogenic microorganisms is called infectious hypopyon. Infectious hypopyon is an important feature of endophthalmitis. There are four types of hypopyon based on the causative agent: fungal, bacterial, oomycete, and viral. Fungal eye diseases are a highly prevalent entity among infectious ocular disorders. In a retrospective study of 1,529 eyes that underwent lamellar keratoplasty at Shandong Eye Institute from 1993 to 2008, Qu and Xie demonstrated that infectious keratitis was the leading indication for the procedure, and fungal pathogens accounted for more than half of all infectious etiologies. For this reason, hypopyon secondary to fungal infection is presented first in the section on infectious causes in this review, intended to provide a more thorough clinical understanding for frontline ophthalmic practice (4).

#### 3.1.1 Fungal hypopyon

For exogenous infectious endophthalmitis secondary to fungal corneal ulcers, hypopyon presents in the vast majority of cases. This entity disproportionately affects rural populations with a history of vegetative ocular trauma. Delayed diagnosis at primary care facilities, inappropriate corticosteroid administration, and infection with fluconazole-resistant fungal strains are major risk factors for progression from superficial corneal ulcer to intraocular involvement (5). Fungal hypopyon often has a subacute clinical course, meaning it evolves and worsens over days to weeks (6). Needle aspiration diagnosis alone often does not yield positive results and usually requires vitrectomy for confirmation. A crucial diagnostic hint in fungal cases is that intraocular inflammation is clumpy or granulomatous. The hypopyon has that “mound-like” appearance due to the pathogenic properties of the fungus and the physical properties of the exudate. Specifically, the localized invasiveness and cohesive growth of fungal hyphae (7) mean that the fungal infection will establish localized foci in either the cornea or the anterior chamber. The resulting inflammatory response will therefore remain largely localized to that focus. Consequently, the exudate composed of fungal elements, necrotic tissue debris and fibrin will tend to accumulate in one place rather than diffuse uniformly throughout the chamber. The physical properties of the exudate also play a key role. In fungal infections, the inflammatory exudate is a combination of proteins, dead tissue, and fungi. This mixture is more viscous and less mobile than that of the bacterial infection. As a result, it does not produce a level, layered collection (hypopyon) in the inferior anterior chamber like bacterial infections do. Instead, as a result of both the effect of gravitation and the inert effect created by the local inflammatory focus, it assumes a heaped-up “mound-like” morphology (8). Clinically, fungal endophthalmitis typically presents with mild ocular pain and conjunctival hyperemia—both generally less severe than in bacterial endophthalmitis—accompanied by scant or absent ocular discharge (9). This clinical profile stands in marked contrast to that of bacterial endophthalmitis, which is classically characterized by pronounced ocular pain, prominent ciliary injection, and abundant purulent discharge (10). Hypopyon is less prevalent in fungal endophthalmitis than in bacterial endophthalmitis (11, 12), a difference accounted for by the more acute onset and more robust exudative inflammatory response that characterize bacterial intraocular infection (13). With respect to macroscopic appearance, fungal hypopyon typically displays a greenish-brown discoloration, may contain pigment granules, and has a characteristically viscous consistency. By contrast, bacterial hypopyon is classically pale white and thin in texture (14, 15). The two entities also diverge in their pattern of intraocular inflammatory involvement. Bacterial endophthalmitis usually demonstrates a diffuse distribution of inflammatory infiltrates, whereas fungal endophthalmitis tends to present with a more focal, clustered pattern (16). Furthermore, fluffy or beaded opacities within the anterior chamber or vitreous cavity are commonly observed in fungal endophthalmitis (17, 18). The following is a summarized comparison of the clinical diagnostic differences between fungal and bacterial endophthalmitis (Table 1).

**Table 1:** Differential diagnosis: fungal vs. bacterial endophthalmitis.

Differential dimensions	Bacterial endophthalmitis	Fungal endophthalmitis
Ocular pain & hyperemia	Marked ocular pain with pronounced ciliary injection, often accompanied by eyelid edema and increased ocular discharge	Mild ocular pain (8)and conjunctival hyperemia is typically milder than in bacterial infections, with scant or absent discharge (9)
Hypopyon	Common presentation (acutely manifests within days in −80% of postoperative bacterial endophthalmitis cases), (11)…Presents as a uniform, white level with a relatively thin, less viscous consistency (18)	Relatively uncommon (12); if present, can appear “green-brown” or may contain pigment granules; typically viscous in consistency (15)
Pattern of inflammation	Inflammation demonstrates a diffuse distribution, with inflammatory cells evenly disseminated throughout the anterior chamber and vitreous cavity (18)	The inflammation presents in a clumped distribution; flocculent or “string-of-pearls” opacities may be observed in the anterior chamber or vitreous cavity (15)

##### 3.1.1.1 The pathogenic sequence can be delineated as follows

###### 3.1.1.1.1 Fungal invasion and proliferation

The process of the binding of fungal conidia to intraocular tissues starts with the use of surface adhesin proteins (Figure 1). After they attach to a surface, the conidia take up water and grow hyphae within the eye itself, especially in the vitreous body (19). The extension of hyphae into retinal and choroidal tissues occurs via mechanical pressure and the secretion of enzymes degrading the extracellular matrix. Both of these enhance the invasive capacity of hyphae (20). Certain fungi (Candida albicans, Aspergillus) may also form biofilms on the surface of intraocular tissues. The biofilm matrix protects the fungi against immune clearance by the host and decreases their permeability to antifungals considerably. Biofilm-associated resistance is a major cause of therapeutic failure (21).

**Figure 1:**
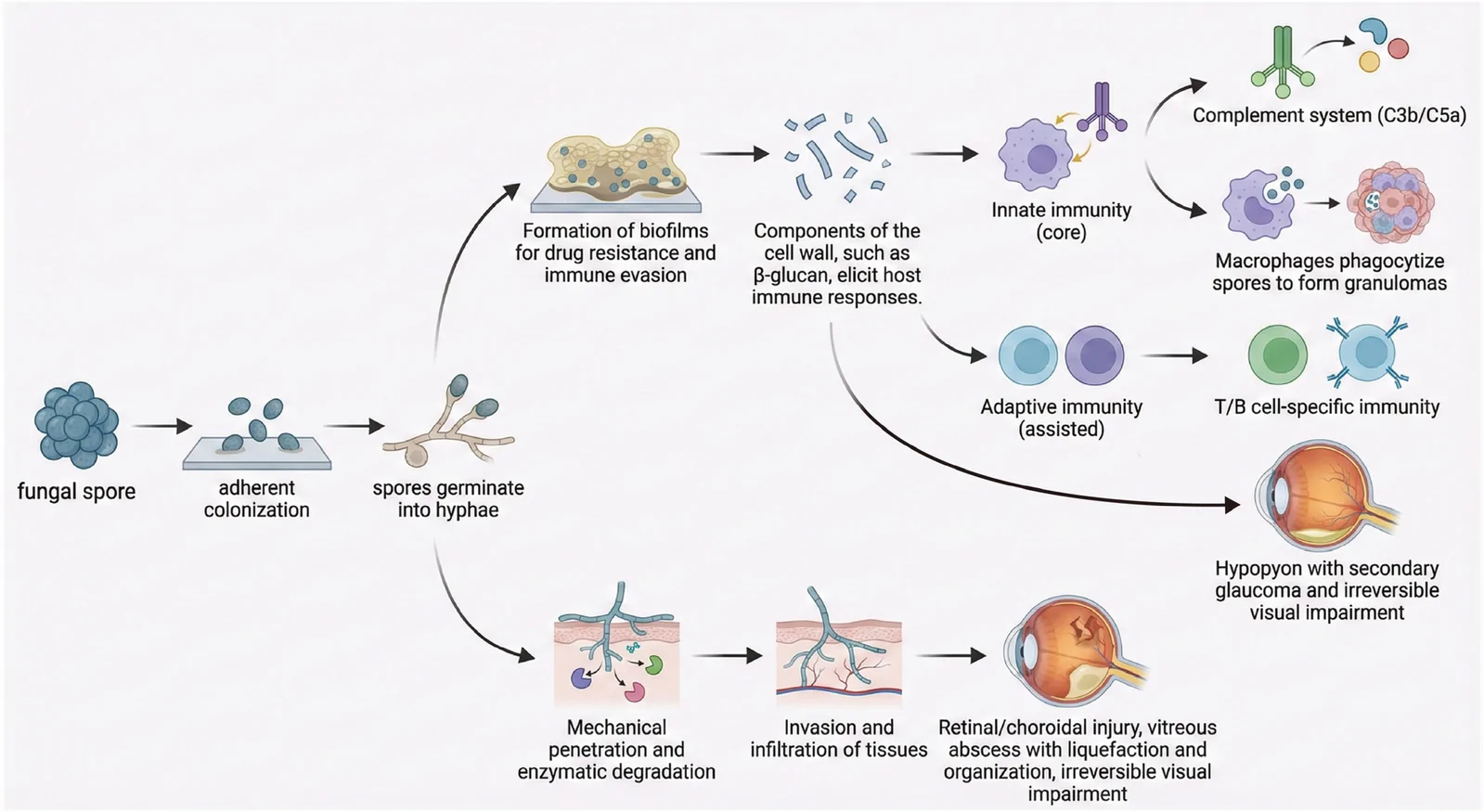
Pathological progression of fungal intraocular infection spanning fungal colonization, immune activation and irreversible ocular structural injury. This schematic illustrates the full pathogenic pathway of fungal endophthalmitis: fungal spores first adhere to ocular surfaces and germinate into hyphae, with biofilm generation mediating antifungal drug resistance and immune escape. Fungal cell wall β-glucan triggers innate immune complement cascades, granuloma formation and downstream T-/B cell adaptive immunity. Concurrent hyphal tissue penetration via mechanical force and enzymatic degradation causes retinal and choroidal destruction, vitreous abscesses, hypopyon, secondary glaucoma and permanent vision loss.

Candida (Yeast-like Fungi) is a group of organisms that produce a lot of β-glucans which are a component of their cell wall. This molecule is a powerful PAMP that can induce a harmful immune response from the host (22).

###### 3.1.1.1.2 Host immune and inflammatory response

Activation of Innate Immunity (The Core Defense Mechanism)

Activation of the complement system, in turn, leads to the generation of C3b and C5a (8) (Table 2). Monocytes and macrophages constitute the foundational line of defense for early recognition and phagocytic killing, whereas neutrophils assume a more prominent effector role during the hyphal stage; both cell types rely on the NADPH oxidase-driven respiratory burst to mediate antifungal defense (23, 24). The function of neutrophils in established fungal infections is often more restricted (25).

2. Involvement of Adaptive Immunity (Facilitates Chronic Clearance)

**Table 2:** Host immune responses to infections caused by different pathogens.

Immune type	Key feature	Fungal infection	Bacterial infection	Viral infection
Innate immunity	Triggering ligands	Cell wall components (β-glucan)	Cell wall components (LPS, peptidoglycan)	Viral surface antigens
	Complement system	C3b opsonization C5a chemotaxis	C3b opsonization C5b-9 forms MAC	Barrier disruption upon overactivation
	Effector cells	Monocytes Macrophages Neutrophil	Neutrophils Macrophages	Macrophages,NK cells
Adaptive immunity	Cellular mediators	T lymphocytes	T lymphocytes; B cell	CD8⁺ T cells; B cells
	Key cytokines	IFN-γ, IL-17	IFN-γ; IL-1, IL-6, TNF-α	IFN-γ, IL-17
	Overall reaction	Facilitates chronic pathogen clearance	Innate immunity dominates	Cytolysis; secondary inflammatory damage

The adaptive immunity response is primarily mediated by T lymphocytes or T cells. Th1 cells particularly secrete interferon-gamma (IFN-γ) (26). At the same time, Th17 cells secrete a cytokine interleukin-17 (Il-17) which plays vital function to get neutrophils to the site of infection. Thus, Il-17 amplifies the early innate inflammatory response (27).

###### 3.1.1.1.3 Tissue damage: a pattern of hyphal invasion and chronic fibrosis

1. Direct Fungal Damage: Mechanical Hyphal Penetration

The direct invasion of tissues is primarily a mechanical phenomenon involving fungal hyphae. This abnormal growth can cause severe structural issues with retinal detachment and choroidal hemorrhages (28). Fungi release diverse proteolytic and degrading enzymes, which attack the structural elements of the vitreous, causing its liquefaction. The process not only helps invade tissue but also frees up nutrients from the decaying matrix that fungi utilize to support further growth and tissue invasion (29).

2. Indirect, Inflammation-Mediated Damage

Chronic inflammation leads to thickening of the vitreous. As a result, formation of fibrotic bands subsequently pulls on the retinal surface that could lead to tractional retinal detachment—a serious vision threatening late complication (28). At the same time, aggregation of fungal hyphae and inflammatory cells in the anterior chamber leads to hypopyon. To be sure, this exudate is thick and tenacious, we can see a mound form, unlike the liquid hypopyon seen in bacterial infections. This thick fluid can block the trabecular meshwork, preventing aqueous outflow and causing secondary glaucoma (28). Because the problem progresses slowly, patients wait a long time before getting checked out. The ongoing destruction of retinal photoreceptors caused by the fungi, along with fibrosis resulting in scarring, often leads to vision loss. As a result, patients may have lasting visual field defects even if the infection is successfully eradicated (30).

##### 3.1.1.2 Diagnostic approach

(I) Advances in ophthalmic imaging have substantially refined the diagnostic evaluation of hypopyon-associated ocular diseases, enabling non-invasive, high-resolution characterization of anterior segment inflammation and pathogenic features (31) (Figures 2–4). Multiple diagnostic modalities are currently applied to identify the etiology of infectious hypopyon: PCR test for intraocular fluid presents excellent potential for quick diagnosis of endophthalmitis. Ophthalmologists might take aqueous humor and vitreous samples.
  1. Aqueous Humor: The liquid is of 0.3 mL each and gets completely displaced within 100 min. Eyes can be sampled via anterior chamber paracentesis (about 0.1 mL).
  2. Aqueous Humor: This is material that has a gel-like consistency and a volume of 4 mL approximately. It exists from birth and is thought to not regenerate. Needle aspiration (0.2–0.3 mL) or through a vitrectomy may be used for sampling (6).
(II) *In Vivo* Confocal Microscopy (IVCM) (32): IVCM allows imaging at 1 μm resolution, permitting direct observation of hyphae or spores in the corneal bed. Concentrating examination notably along the ulcer edge can improve diagnostic yield. Key features you should know about the method are it non-invasive and rapid, but it cannot identify the specific fungal organism.
(III) Corneal Scraping with Giemsa Stain (33): Tissue sampling from the area of incipient lesion is performed using a scalpel. After staining, fungal elements are noted under the microscope. Key features: economical, quick results (1–2 h), but requires skilled sampling technique to prevent misclassification as false negatives due to low sample size.
(IV) Fungal Culture with species identification and antifungal susceptibility testing refers to the solid media inoculated with the specimen. The results of culture are highly speciﬁc and it is thus considered the gold standard in the diagnosis of fungal keratitis (34). Blood agar and Sabouraud dextrose agar are the commonly used media, incubated at 37 °C and 22 °C–25 °C, respectively, for at least one week (35). The basic identification is done through colonial features and microscopic hyphal morphology (36). Antifungal susceptibility testing is done by broth microdilution method (37).
(V) Anterior Segment Optical Coherence Tomography (AS-OCT): This imaging technique allows for the measurement of corneal thickness, infiltrate thickness, and infiltrate width with an impressive vertical resolution of 5 microns. It is vital for diagnosing of any associated findings such as endothelial plaques and hypopyon (38). Slit-lamp biomicroscopy has long served as the standard modality for the diagnosis, monitoring, and management of anterior segment inflammation. However, this approach is inherently limited by its subjective nature and dependence on patient cooperation, which presents particular challenges in individuals with photophobia or limited compliance, most commonly children (39). Anterior segment optical coherence tomography (AS-OCT) is gaining widespread recognition as a non-invasive, more objective imaging alternative (40). Capable of detecting inflammatory cells within the aqueous humor, AS-OCT supports reproducible serial measurements and is less susceptible to patient-derived bias (39). The integration of artificial intelligence has further strengthened this advantage: fully automated deep learning algorithms can autonomously segment the anterior segment, localize the region of interest, and quantify hyperreflective inflammatory foci without manual input, demonstrating strong concordance with SUN clinical grading and enabling detection of subclinical inflammation that is missed on slit-lamp examination. Beyond cell counting, AI-enabled en face OCT reconstruction also permits precise volumetric quantification of keratic precipitates, providing an objective biomarker for longitudinal monitoring of disease activity and treatment response. The initial clinical application of AS-OCT for this indication was first reported by Agarwal et al. in 2009, who demonstrated that the technique enables detection of anterior chamber inflammatory cells along the corneal endothelium even in the presence of corneal edema or infiltration. In 2025, Bellchambers et al. further extended the evidence base, revealing that AS-OCT can not only objectively quantify intraocular inflammation, but also differentiate pigment cells from inflammatory cells (41).

**Figure 2:**
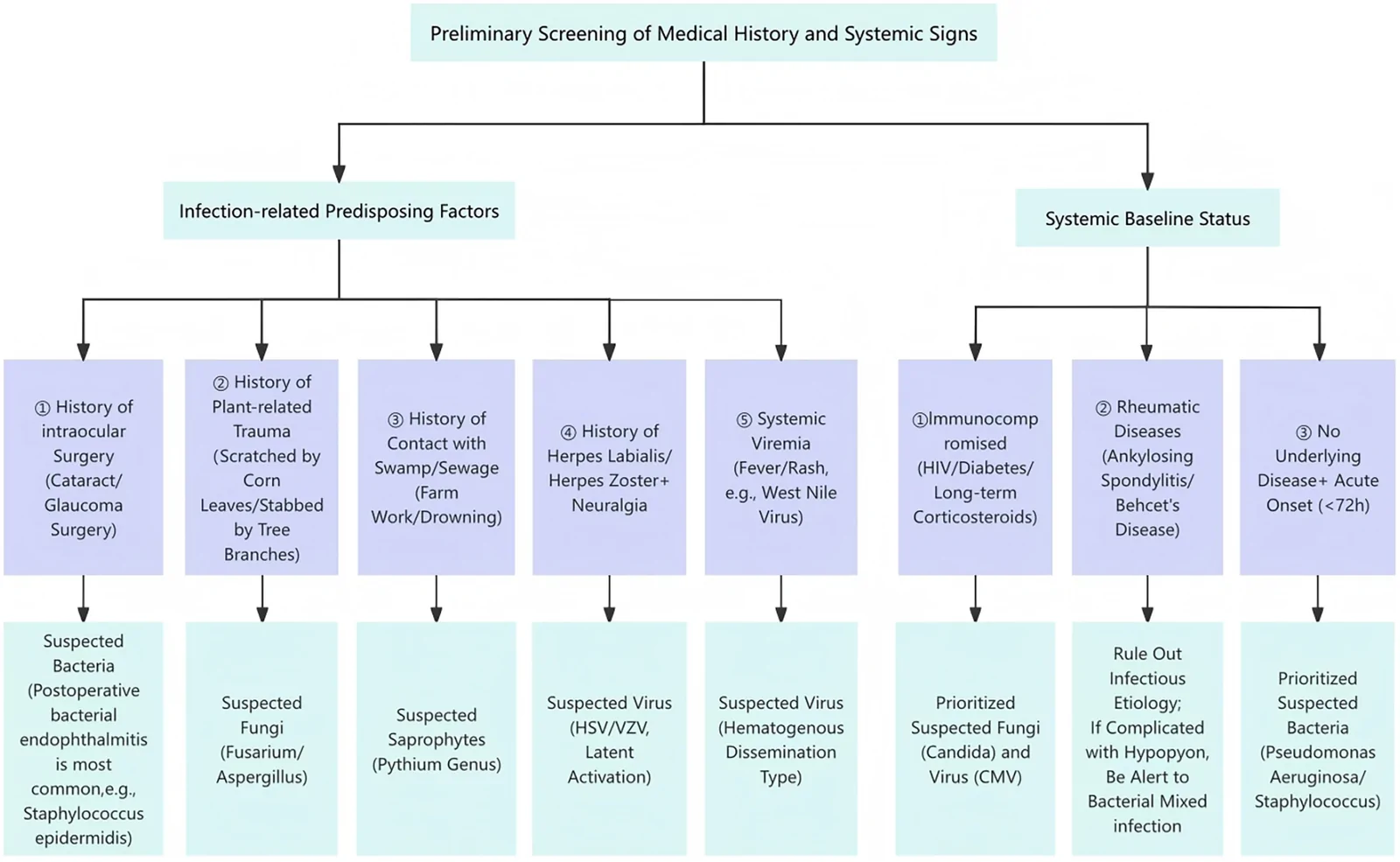
Two-arm clinical preliminary screening flowchart for infectious endophthalmitis etiology differentiation via medical history and systemic baseline assessment. The left branch stratifies probable bacterial, filamentous fungal, oomycete, viral hematogenous and latent viral pathogens according to surgical history, plant-related trauma, swamp exposure, herpetic episodes and systemic viremia records. The right branch evaluates host immune status, rheumatic comorbidities and disease onset timing to prioritize high-risk pathogens, flag mixed infection risk and guide subsequent targeted laboratory testing.

**Figure 3:**
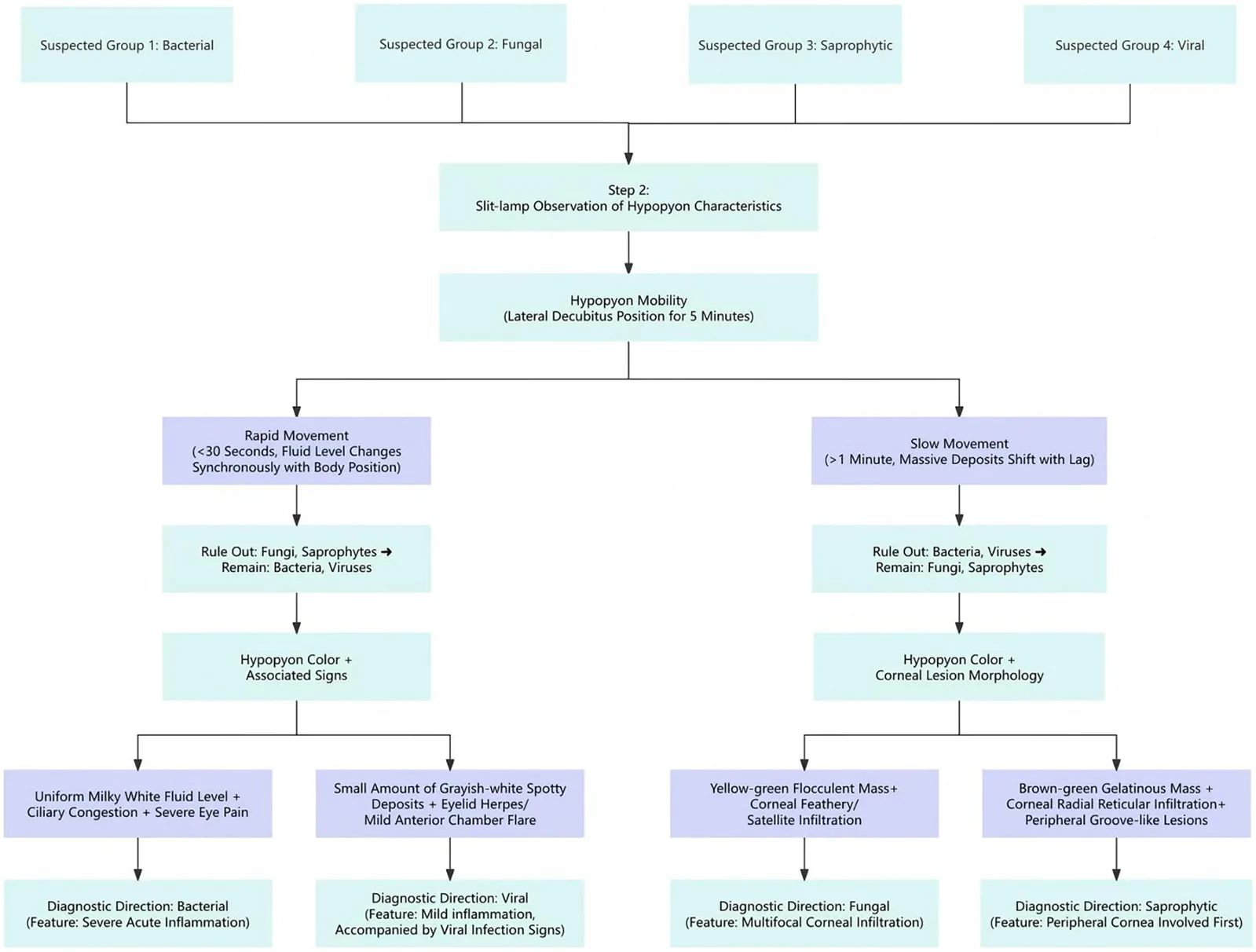
Stepwise slit-lamp diagnostic algorithm differentiating four pathogen categories by hypopyon kinetic traits, color and concurrent ocular surface morphology. After preliminary pathogen grouping, clinicians grade hypopyon positional shift velocity during 5 min of lateral decubitus positioning (Bedside positional-shift grading of hypopyon. This is an author-proposed, unvalidated protocol; its diagnostic accuracy and inter-observer agreement have not been formally evaluated). Rapid hypopyon movement narrows candidates down to bacterial and viral etiologies, while sluggish sediment motion points toward fungal and oomycete infection. Supplementary inspection of pus hue plus corneal lesion architecture yields definitive preliminary diagnostic orientation for each pathogen subtype.

**Figure 4:**
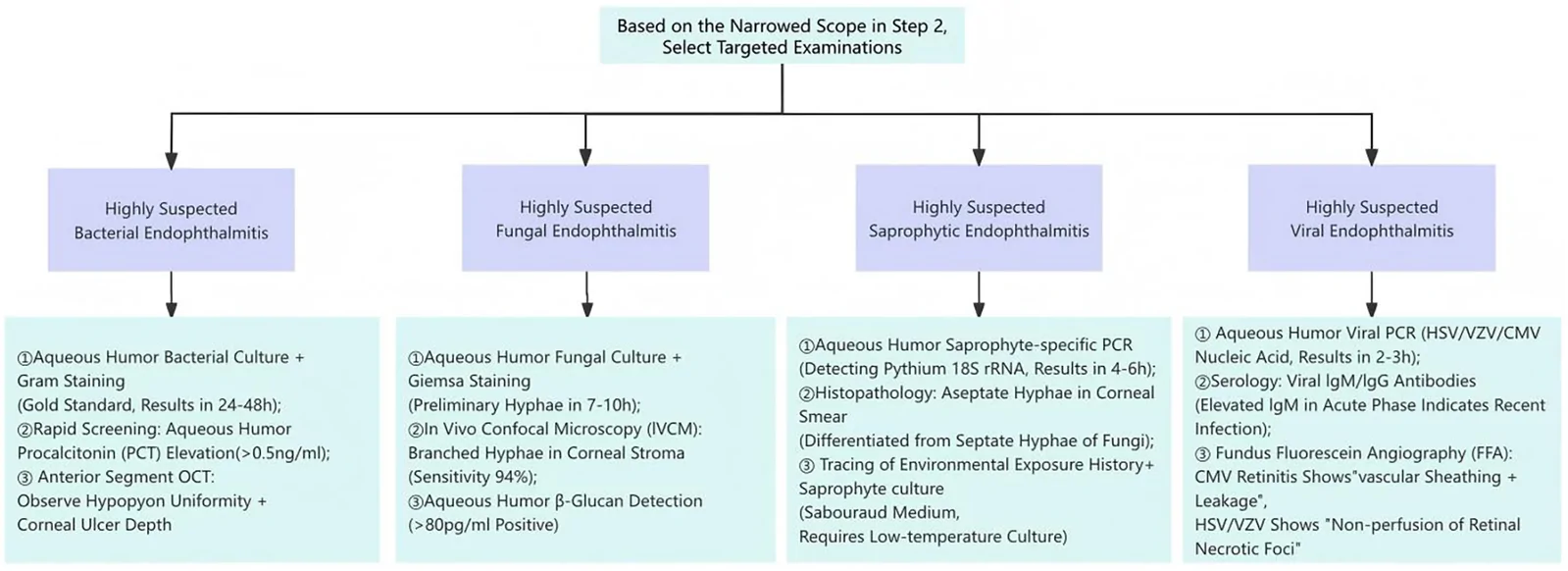
Targeted laboratory workup framework tailored for four pathogen subgroups after clinical phenotypic triage. Diagnostic testing bundles are customized for high-probability bacterial, fungal, oomycete (Pythium) and viral intraocular infections. Each branch lists gold-standard microbial culture, rapid molecular PCR, specialized ophthalmic imaging, serologic assays and biomarker detection.

#### 3.1.2 Bacterial hypopyon

The condition usually develops within days of the event. The intraocular inflammation is usually diffuse in nature. In cases of Propionibacterium acnes endophthalmitis, sampling the white plaques on the posterior lens capsule yields the highest chance of a positive culture. Chronic post-cataract endophthalmitis that responds to topical corticosteroids but recurs when tapered is highly suggestive of a bacterial etiology, most likely P. acnes (6).

The treatment principles for bacterial endophthalmitis are similar to those for fungal endophthalmitis. The importance of early antibiotic (e.g., vancomycin) administration to slow disease worsening and improving the efficacy of treatment and postoperative quality of life are stressed. In accordance with the clinical practice guidelines issued by the European Society of Cataract and Refractive Surgeons (ESCRS), vancomycin and ceftazidime (or an alternative third-generation cephalosporin) are recommended as first-line antibiotic agents for intravitreal administration. The standard recommended dosages are 1 mg of vancomycin delivered in 0.1 mL volume and 2.25 mg of ceftazidime delivered in 0.1 mL volume. Management strategy is laid upon three cornerstones. Treatments commence with suitable pharmacotherapy. Then it is followed by proper targeted therapy. Lastly, surgery should be carried out when the condition demands it. High-dose early intravitreal antibiotic therapy is the keystone. In extreme situations that fulfil certain criteria, it is most important to have immediate pars plana vitrectomy with intravitreal injection of antibiotics (IVIAs). Systemic and topical drug administration is limited by multiple ocular barriers, making it difficult to achieve effective therapeutic concentrations in the vitreous cavity. Intravitreal injection is the current standard of care for all types of infectious endophthalmitis (42). Intravitreal antibiotics alone fail to achieve complete intraocular bacterial eradication in a subset of eyes. For severe cases, adjunctive pars plana vitrectomy (PPV) improves clinical outcomes (13); PPV enhances antibiotic penetration by removing the vitreous body (a potential medium for bacterial growth), reducing bacterial load and toxins, and improving intravitreal circulation; it also increases the positive rate of microbial cultures, thereby facilitating targeted antimicrobial therapy (43).

##### 3.1.2.1 The pathogenic sequence can be delineated as follows

###### 3.1.2.1.1 Bacterial invasion and proliferation

Bacteria attach to intraocular structures through surface adhesin proteins, leading to biofilm formation (44) (Staphylococcus epidermidis produces biofilm that confers high resistance to antibiotics and host immune clearance) (45) (Figure 5).

**Figure 5:**
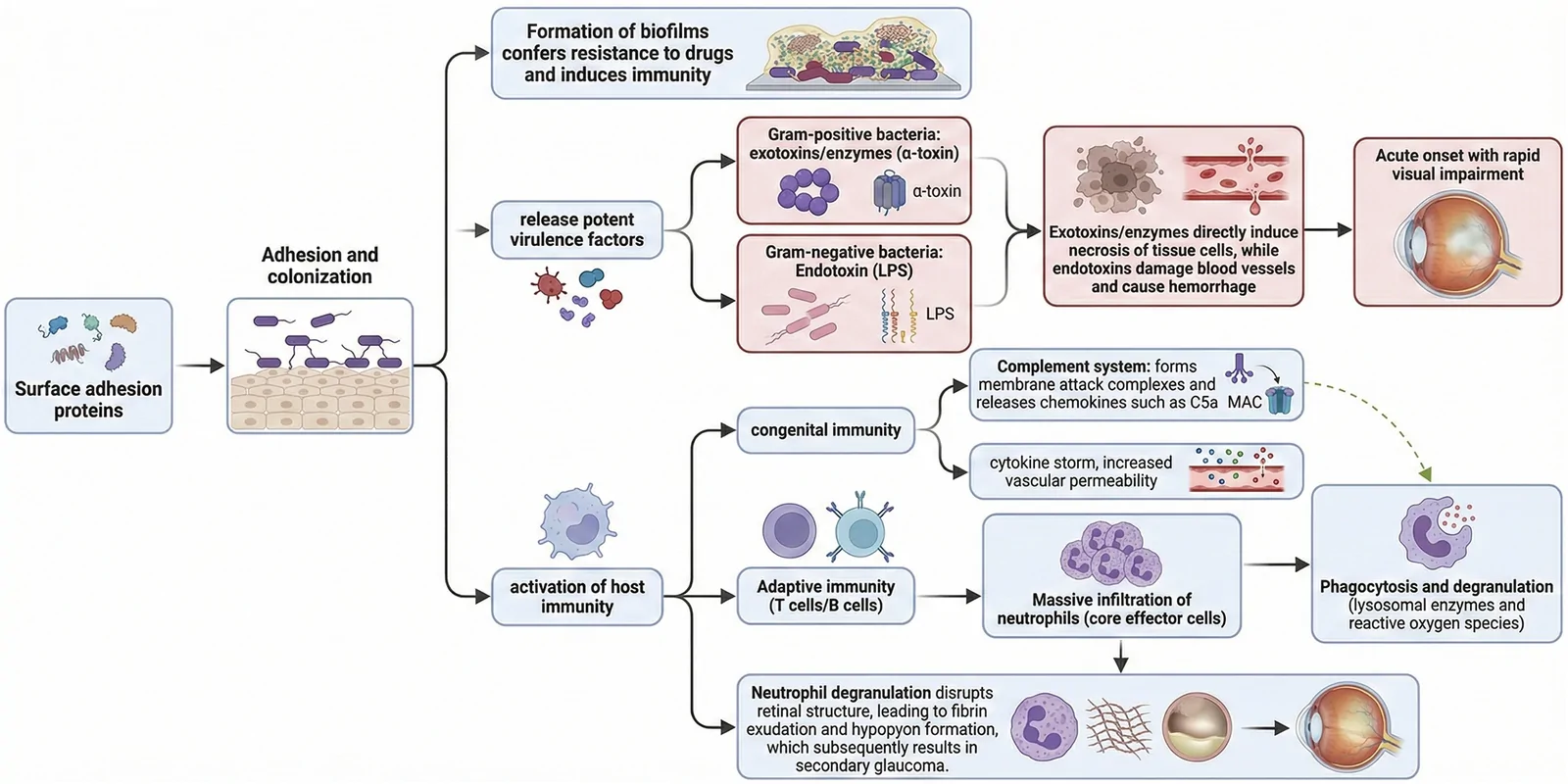
Multistep pathogenesis schematic of bacterial endophthalmitis driven by microbial virulence mediators and host inflammatory cascades. Bacterial surface adhesion proteins mediate corneal colonization; secreted exotoxins from Gram-positive strains and lipopolysaccharide endotoxin from Gram-negative pathogens directly induce corneal necrosis and vascular hemorrhage. Innate complement signaling plus T- and B-cell adaptive immunity recruit abundant neutrophils; granulocytic degranulation breaks down retinal architecture, generating fibrin exudate, hypopyon, secondary glaucoma and abrupt vision deterioration.

###### 3.1.2.1.2 Host immune response

Bacterial components such as lipopolysaccharide or peptidoglycan can activate the alternative complement pathway, generating C3b and C5b-9 that facilitate opsonization and formation of a membrane attack complex (MAC), respectively, thus causing direct bacteriolysis. At the same time, chemotactic factors like C5a recruit neutrophils, which eliminate pathogens via phagocytosis and degranulation (release of lysosomal enzymes and reactive oxygen species) (46, 47). Excessive degranulation, however, causes collateral damage to surrounding healthy tissues, especially the retina (48). Cytokine Storm: Macrophages and retinal pigment epithelial cells secrete pro-inflammatory mediators that recruit inflammatory cells, increase vascular permeability and propagate an inflammatory cascade (49–51). Th1 cells strongly activate and enhance the bactericidal activity of macrophages by secreting IFN-γ, an important cell-mediated defence axis against intracellular pathogens (26). Nevertheless, given the limited intraocular penetration of antibodies, the overall contribution of adaptive immunity is less pronounced than that of innate immunity.

###### 3.1.2.1.3 Tissue injury: a dual mechanism of direct bacterial destruction and inflammation-mediated damage

Bacteria primarily damage tissues through the rapid release of exotoxins and endotoxins (52). This is a pathogenic strategy distinct from the use of enzymes to facilitate damage by fungi. Exotoxins (α-toxin) and enzymes (coagulase) produced by Gram-positive bacteria mainly Staphylococcus aureus and S. epidermidis damage intracellular cells directly and favour inflammation's spread (53). Gram-negative bacteria such as Pseudomonas aeruginosa, Escherichia coli secrete endotoxins (lipopolysaccharide, LPS) which activate immune cells of the host, thus inducing an over-the-top inflammatory response (54). Additionally, the pigments that the pathogenic bacteria generate influence the hypopyon coloration directly. This identifiable trait can act as a useful clinical marker for differentiating among the causative organisms (Table 3).

**Table 3:** Changes in the color of hypopyon caused by different pathogenic bacteria.

Causative pathogen	Characteristic hypopyon color/appearance	Pathogenesis & clinical response
Gram-positive bacteria	Creamy white/yellow; viscous with defined level	Classic suppuration; dense pus from neutrophil influx
Gram-negative bacteria	Yellow-green/white; watery pus	Green pigmentation (pyocyanin); Endotoxin-driven severe inflammation

The secondary injury that is caused by inflammation occurs when neutrophil degranulation causes the release of reactive oxygen species and proteases like matrix metalloproteinases which degrades retinal photoreceptors and also the pigment epithelium. Enhanced blood vessel leakage causes discharge of fibrin leading to the formation of a hypopyon. This further blocks the outflow of aqueous humor and raises pressure inside the eyeball.

##### 3.1.2.2 Diagnostic approach

###### 3.1.2.2.1 Etiological testing (gold standard)

Vitreous specimens should be preferentially collected (Figures 2–4). The yield of Vitrectomy samples is positive in about 90% of cases, which is much higher than vitreous tap (50%–70%) (55).

Isolates need to be cultured on the medium like blood agar or chocolate agar at 37 °C. Most bacteria, such as coagulase-negative staphylococci and streptococci, form discernible colonies within a span of 24–48 h (Table 4).

**Table 4:** Microbiological profile of common bacterial pathogens.

Bacterial species	Culture & observation period	Colonial morphology
Staphylococcus spp.	18–24 h	On blood agar, they form circular, convex, smooth colonies with entire margins Coagulase-negative staphylococci typically appear white and are non-hemolytic
Streptococcus	18–24 h	Circular, convex, gray-white colonies on blood agar; hemolysis patterns vary by species (α, β, or γ)

About 70%–90% of cultures of postoperative bacterial endophthalmitis are positive. It is recognized that Gram-positive cocci are present in 95% of the culture-positive specimens of acute post cataract endophthalmitis (6).

###### 3.1.2.2.2 Molecular diagnostics

Pan-bacterial 16S rRNA gene PCR (conventional or real-time): One of the main advantages of this test is quick turnaround time of results (2–3 days), high sensitivity, and unaffected by prior antibiotic exposure. Particularly useful for detecting pathogens in culture-negative cases (56). According to a study, about 20%–30% of suspected bacterial endophthalmitis are culture-negative. However, this method can detect culture-negative cases making it powerful (57). It cannot observe the fungal organism and needs a species-specific primer design for certainty in identification (58). Mohaddese Hajinezhadian et al. developed a SYBR Green-based multi-panel real-time PCR assay capable of detecting six endophthalmitis-associated bacterial species from vitreous samples within 4 h. Notably, this assay retains diagnostic utility for pathogen identification even in a proportion of culture-negative clinical cases (59). Yang et al. established a highly sensitive and specific detection platform harnessing multiplex crRNAs in the CRISPR-Cas12a system. This platform enabled detection of genomic DNA from four common fungal pathogens causing corneal infections in as little as 32 min. Furthermore, the assay can be performed using simulated tear samples as input, which substantially enhances its portability and biosafety profile. Multiplex crRNA can offer greater rapidity, convenience, and sensitivity compared with conventional methods such as PCR (60). Infectious endophthalmitis generally shows no WBC abnormalities on CBC (though counts may be elevated in severe infection). While traditional aqueous humor culture yields positive bacterial or fungal growth in only a minority of cases, nanopore targeted sequencing, a next-generation molecular diagnostic modality, achieves a significantly higher pathogen detection rate (90.91% vs. 38.18% for culture) with a much shorter turnaround time (1.13 vs. 2.67 days on average). It enables simultaneous identification of bacteria, fungi, and polymicrobial infections, providing more reliable etiological evidence for differential diagnosis (61).

###### 3.1.2.2.3 Staining examination

Gram-positive cocci (e.g., staphylococci): Purple, spherical cells typically arranged in clusters.

Gram-negative rods (e.g., Pseudomonas aeruginosa): Pink/red, rod-shaped bacilli.

When examining, it is critical to not mistake iris pigment granules for Gram-positive cocci as iris pigment granules are high refractile “soccer-ball” structure (62).

###### 3.1.2.2.4 Metagenomic next-generation sequencing (mNGS)

Metagenomic next-generation sequencing (mNGS) delivers a high diagnostic yield for intraocular infectious diseases, owing to its distinctive detection strategy that enables simultaneous acquisition of millions to billions of nucleic acid sequences for the identification of diverse microorganisms. Accordingly, mNGS demonstrates superior sensitivity compared with conventional detection methods, and achieves optimal diagnostic performance when the proportion of host DNA is low or pathogen sequences are enriched. However, given its ultra-high sensitivity, false-positive results represent a critical concern; thus, the implementation of negative controls for reagents and specimen containers is strongly recommended (63). The first report of mNGS applied to clinical diagnostic practice was published in 2014. This seminal study was the first to demonstrate that mNGS could generate clinically actionable information and render a definitive clinical diagnosis, thereby facilitating appropriate patient management and validating the feasibility of this technology in clinical settings (64). Since it requires only a minute volume of intraocular fluid, mNGS is recognized as an efficient and safe diagnostic modality. Currently, mNGS has been integrated into clinical diagnostic workflows and is specifically utilized for infections that are refractory to identification by routine diagnostic tools such as polymerase chain reaction (PCR) (63). Accumulating diagnostic accuracy studies have systematically evaluated its performance against established reference standards, reporting detailed metrics including sensitivity, specificity and predictive values. A recent meta-analysis synthesizing data from 21 studies involving 1,219 eyes demonstrated that mNGS achieved positive rates ranging from 10% to 94% across diverse ocular infection phenotypes, with reported sensitivity of 15%–100% and specificity of 12%–100%; the technology identified a broad pathogen spectrum at both genus and species levels, with viruses being the most frequently detected category (reported in 15 of 21 included studies), followed by bacteria (14/21), fungi (10/21) and parasites (6/21) (65). Beyond pathogen identification, mNGS also enables detection of antimicrobial resistance (AMR)-associated genes and mutations, which may inform individualized treatment selection. A multicenter prospective study of 488 patients further corroborated these findings using cell-free DNA (cfDNA)-based mNGS in 152 vitreous humor (VH) and 336 aqueous humor (AH) samples, with final composite clinical diagnosis as the reference standard: among the enrolled cohort, 288 patients were confirmed to have infectious ocular disease, 161 had non-infectious conditions, and 39 had indeterminate final diagnoses, and mNGS performed slightly better in VH samples, with a sensitivity of 92.2% and overall agreement rate of 81.3%, compared with 85.4% sensitivity and 75.4% overall agreement rate in AH samples (66). The multicenter randomized controlled OPTICS study further supported the clinical effectiveness of mNGS-aided diagnosis. Among the 21 randomized participants, one was lost to follow-up, leaving 20 evaluable patients (9 in the mNGS-guided cohort and 11 in the standard-of-care control group). At the 4-week follow-up, 88.9% of patients in the mNGS group achieved clinical improvement, a rate markedly higher than the 63.6% observed in the control group. In terms of safety, only 3 adverse events unrelated to the study intervention were reported during the entire follow-up period (67). Despite its strengths of high flexibility and exceptional sensitivity, mNGS performance is considerably affected by multiple factors, including host DNA abundance and environmental pathogen sequences. At present, it is not routinely recommended as a first-line diagnostic test, but rather serves as an adjunctive tool or a complement to first-line diagnostic assays.

#### 3.1.3 Hypopyon caused by Pythium infection

Giant cell producing parasitic aquatic oomycete Pythium insidiosum of the family Pythiaceae is found in aquatic habitats of tropical, subtropical and temperate regions. Clinically and morphologically, this organism causes corneal damage that resembles fungal keratitis, leading to its frequent classification as a “fungus-like” pathogen.

There has been a rise in the cases of Pythium keratitis. Because it can mimic fungal keratitis, a differential diagnosis is essential. The primary diagnostic method is the culture on blood agar or other nutrient media which appears as creamy white colonies and produces motile zoospores which is characteristic feature. The leaf incarnation method can be used for further confirmation. Given the limited lab diagnostic options and delayed growth of culture, there is a recent trend towards molecular diagnostics. These consist of the polymerase chain reaction (PCR), enzyme-linked immunosorbent assay (ELISA), and immunodiffusion assays (68). Due to its high sensitivity and specificity, apart from positive culture with zoospore formation, PCR tests are the most important diagnostic modalities, as they typically identify species-specific sequences of the DNA (69, 70).

##### 3.1.3.1 The pathogenic sequence can be delineated as follows

###### 3.1.3.1.1 Initiation of infection: zoospore localization and adhesion

The contractile zoospores of Pythium are typically found in stagnant water bodies like swamps (Figure 6). They are known to move towards the wounds on the fish skin or eye through chemotaxis. When they come into contact with exposed tissue, they encyst and firmly adhere (71).

**Figure 6:**
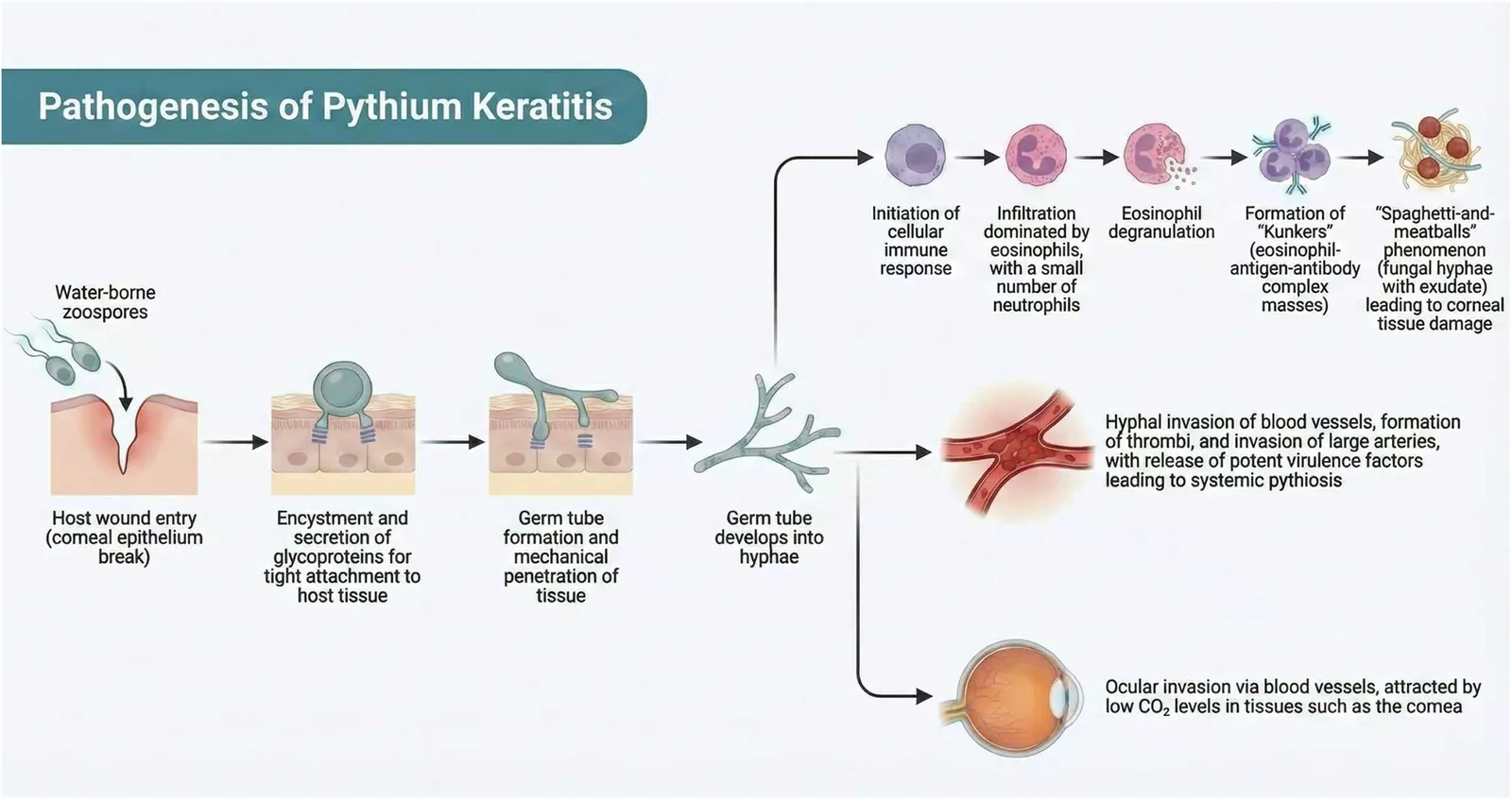
Progressive pathogenic cascade of pythium keratitis following water-borne zoospore corneal epithelial invasion. Aquatic zoospores access the stroma through corneal epithelial defects, encyst and anchor to host tissue via glycoprotein secretion before germ tubes mechanically penetrate stromal layers and mature into invasive hyphae. Eosinophil-predominant inflammation generates characteristic antigen-antibody aggregates; vascular hyphal invasion triggers thrombus formation, local corneal necrosis and hematogenous spread that can cause systemic pythiosis.

###### 3.1.3.1.2 Invasion mechanism: from encystment to tissue penetration

After the zoospores adhere, they secrete adhesive glycoproteins to prevent detachment from the host. At the same time, the body temperature of the host is a major stimulus of the cysts.

In response to the heat stimulus, the encysted zoospores germinate producing a germ tube which penetrates. The germ tube enters the host tissue through mechanical force and forms the first structures to invade (68).

###### 3.1.3.1.3 Disease outcomes in different hosts/tissues

Infection manifests in three forms after germ tube penetration. They depend on the host species and the tissue invaded.

1. Localized Infection in Equines

A cell-mediated immune response begins, with heavy infiltration of eosinophils and lesser of neutrophils. In the pathology, eosinophils degranulate and aggregate to the hyphae, resulting in firm caseous masses that are called “kunkers”. When eosinophils attempt to contain the pathogen they release toxic chemicals with an end result of severe damage to the local tissue (68).

2. Systemic Pythiosis (Disseminated Infection)

The hyphae show a distinct preference for large arteries after entering the blood stream, causing vascular invasion and thrombosis. The virulence factor of the pathogen helps in wide dissemination concluding into systemic pythiosis with multi-organ involvement (68).

3. Cutaneous/Ocular Infection (in Humans and other hosts)

Tissue Invasion: The germ tubes cause damage to cutaneous or ocular tissues. Pathogenesis and Damage: The relatively low levels of CO₂ present in tissues attract the hyphae, which results in relatively rapid and severe epithelial and stromal ulceration with a characteristic aggressive behaviour (68).

Based on the work of Bharat Gurnani et al., aggressive treatment must be initiated even before definitive diagnosis when clinical suspicion is high. Pythium insidiosum is morphologically and clinically similar to fungi, but it belongs to the oomycete lineage and lacks ergosterol in its cell membrane, which renders conventional broad-spectrum antifungal agents (e.g., natamycin, itraconazole, voriconazole) ineffective. The microscopic examination of the smear and culture done preferably with a surgical biopsy aids in diagnosis. Upon microbiological confirmation, the therapeutic regimen should be stratified according to ulcer size: for ulcers measuring less than 4 mm × 4 mm, hourly administration of 0.2% linezolid eye drops is recommended; for larger ulcers, hourly combination therapy comprising 0.2% linezolid and 1% azithromycin is indicated. In culture-negative cases, smaller ulcers should be managed with 5% natamycin, whereas larger ulcers warrant combination therapy with natamycin plus either itraconazole or voriconazole (69). In clinically suspicious cases with reticular dot infiltrate, tentacular projections, and peripheral furrowing, before the culture results, the clinician can start the patient on a combination of antifungal and antibacterial (0.2% linezolid and 1% azithromycin) treatment (72, 73). Surgical intervention including keratoplasty or vitrectomy is often needed early and aggressively when necrotic tissue and hyphal masses require removal as medical therapy is often inadequate.

Ongoing clinical evaluation is essential to monitor for disease progression/recurrence and adaptability of treatment strategy based on clinical response. An integrated medical-surgical approach is essential to optimize outcomes in this sight-threatening infection (74). Surgical treatment is the mainstay of management: In cases of corneal infection, penetrating penetrating keratoplasty (PK) and, when indicated for intraocular extension, pars plana vitrectomy (PPV) may be required to excise necrotic tissue and invading hyphae before perforation or extension to the sclera happens (75). If the case progresses to endophthalmitis, a pars plana vitrectomy has to be added to debride the infectious foci. It is important to achieve adequate surgical margins for minimising recurrence risk. Moreover, when it comes to antimicrobial therapy, susceptibility testing should be used to optimize efficacy without causing the use of antifungals.

#### 3.1.4 Viral hypopyon

Some common viruses that can cause infectious diseases include herpes simplex, varicella-zoster, cytomegalovirus, and retroviruses, such as HIV.

Hypopyon in Herpes Zoster Keratouveitis: Herpes zoster ophthalmicus (HZO) occurs due to the reactivation of varicella-zoster virus (VZV) in the ophthalmic branch of the trigeminal nerve. It is well known that the virus can infect any ocular structure. The acute forms of manifest includes blepharoconjunctivitis, epithelial keratitis or anterior uveitis. After the onset of cutaneous rash, the eyelids and conjunctiva involvement occurs typically within 1–2 weeks in HZO patients. Vesicles with eye characteristics appear on eyelids and/or bulbar conjunctiva, often with blepharitis. Unilateral conjunctivitis, with or without petechial hemorrhages, and periorbital edema may be present (76). Cytomegaloviral (CMV) anterior uveitis with hypopyon is a recurrent ocular inflammatory disease that can involve the entire uveal tract. Viral causes make up about 10% of anterior uveitis cases. Condition usually presents as a sudden decrease in vision on one side and usually has a headache on the same side. Mild redness of the eyes is seen in the clinic. As well, there may be slight swelling of the corneal epithelial cells. And, there are small or pale keratic precipitates that are scattered either in the centre or outside. There may be a slight reaction to the front chamber which can be mild with atrophy of the iris. The angle of the anterior chamber of the eye is open, while optic disc appearance and visual fields are generally normal at presentation, although there may be a mild decrease in visual acuity (77). Infection with retroviruses, for example, the HIV, results in a very strong cellular immunodeficiency and alters the immunologic equilibrium of the host. This breakdown in the body allows for fungi from other areas where colonization or infection occurs to spread through the blood later, passing through blood vessels in the eye and causing having endogenous fungal endophthalmitis. The resulting eye symptoms, including hypopyon shape, are classic for fungal endophthalmitis (78), and are entirely consistent.

It is strongly recommended to start antiviral treatment within 72 h of symptoms. Yet patients having complications or those at very high risk of complications, such as those with coexisting ocular involvement and those who are immunocompromised, and who display evidence of active replication (e.g., new lesions) 72 h after lesion appearance should be treated according to European consensus (79).

It is crucial for ophthalmologists to adopt tailored diagnostic and therapeutic strategies based on the variations in hypopyon caused by different types of infectious endophthalmitis (Table 5).

**Table 5:** Clinical characteristics of infections by different pathogens.

Pathogen type	Primary route of infection	Host immune response	Typical tissue injury
Bacterial	Exogenous endogenous	Neutrophil-dominated acute inflammatory storm	Acute necrosis; pigmented hypopyon; secondary glaucoma
Fungal	Exogenous endogenous	Macrophage-dominated chronic granuloma	Chronic fibrosis; retinal tears/detachments
Pythium	Exogenous	Neutrophil infiltration	Corneal radial infiltration; hypopyon (gray-white)
Viral	Reactivation/endogenous spread	Innate immunity + adaptive immunity	Retinal necrosis

##### 3.1.4.1 The pathogenic sequence can be delineated as follows

###### 3.1.4.1.1 Viral replication and intraocular dissemination

1. Ocular Cellular Tropism of Viruses: Adenoviruses and HSV can infect the iris and ciliary body epithelium, causing inflammation in the anterior chamber (anterior uveitis) (Figure 7). Clinically, this manifests as increased anterior chamber cells and the formation of iris synechiae, which are both observed at the slit lamp (80). HSV infections generally display less acute presentation and tend to have a more indolent course that is recurrently seen, with characteristic corneal scarring (81). Hypopyon may develop, characteristically as a sterile, fibrin-rich accumulation, signifying significant inflammation in severe viral anterior uveitis (82, 83).
2. Viral Replication and Dissemination Patterns: When the HSV virus gets into cells it can make copies of itself in those cells within a few hours. This can be seen as retinal pathology in 1–3 days time (84). Their pathogenesis is marked by a rapid cytolytic cycle that can cause considerable tissue damage and inflammation. After the Initial Infection, These Viruses Establish Latency Within Sensory Ganglia. Following the primary infection, these viruses establish lifelong latency within sensory ganglia (e.g., the trigeminal ganglion) (85).

**Figure 7:**
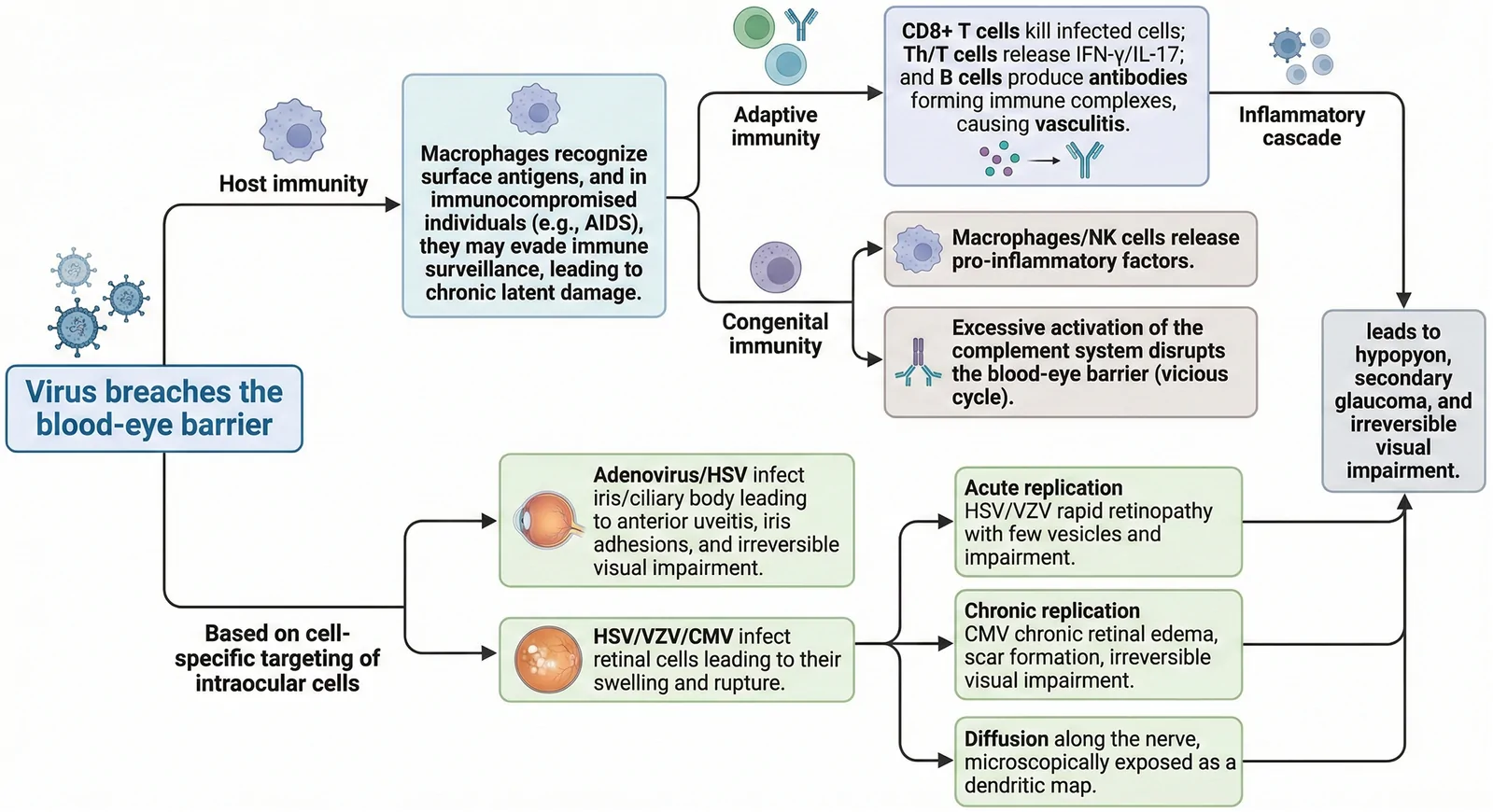
Immune and tissue-level injury mechanisms of viral endophthalmitis after viral traversal across the blood-eye barrier. Intraocular viral colonization proceeds via two routes: immune recognition by tissue macrophages (with viral immune evasion in immunocompromised populations) and selective tropism toward iris, ciliary body and retinal parenchymal cells. Cytotoxic adaptive lymphocyte responses, pro-inflammatory innate immune signaling and excessive complement activation amplify barrier breakdown; acute, chronic and nerve-spread viral replication patterns collectively drive intraocular inflammation, hypopyon, secondary glaucoma and permanent visual impairment.

###### 3.1.4.1.2 Host immune response

The ocular immune privilege generally restricts mild inflammation in the eye (intraocular inflammation) via blood-ocular barrier and anterior chamber-associated immune deviation (ACAID). For this reason it preserves eyesight by restraining needless immune-mediated tissue-damage (86, 87). However, such privilege can also turn into a drawback. This can delay recognition and containment of virulent pathogens or cause an exaggerated dysregulated response when the immune system is virally responding upregulated (88). In that case, proliferation occurs at a site (anterior chamber) with a weak inflammatory response (no intraocular inflammation) (89). The process is often clinically silent, only detected if there is substantial vision loss (89, 90).

1. Innate Immune Response: Resident intraocular macrophages recognize viral surface antigens and release substantial amounts of pro-inflammatory cytokines. This promotes NK cell recruitment to eliminate virus-infected cells; however, excessive activation of this response can damage adjacent healthy cells and exacerbate intraocular inflammation, thereby triggering tissue damage (91, 92).
2. Adaptive Immune Response: Viral antigen-presenting cells activate CD8⁺ T cells, which exert specific cytolysis against infected cells through the secretion of perforin/granzyme. However, the release of cytokines is also strongly activated, thereby triggering a series of inflammatory cascades that cause secondary damage (93, 94). Immune complexes formed by virus-specific antiviral antibodies secreted by B cells and viral antigens may also accumulate in retinal vessel walls, inducing immune complex-mediated vasculitis and further exacerbating vascular damage (95).

###### 3.1.4.1.3 Mechanisms of tissue injury

The early stage features direct viral destruction as the primary engine at work. The virus causes infection and the target cell will swell and burst. When photoreceptors are destroyed, light signals are not converted into neural impulses (96). In the middle or late stages, high levels of IL-1, TNF-α and IFN-γ (cytokine storm). This strong inflammatory environment does not eliminate the infection. It also induces retinal neuron apoptosis and widespread glial scarring, leading to irreversible damage to tissue (93). The disruption of the blood-ocular barrier allows the entry of white blood cells and proteins into the aqueous humor and vitreous body, leading to hypopyon and vitritis (97).

### 3.2 Non-infectious hypopyon

As mentioned before most cases of hypopyon are due to infectious causes, hence infection must be the first consideration in differential diagnosis. Non-infectious causes, especially, drug reactions and autoimmune diseases, represent two different types of entities that have fundamentally different mechanisms of pathogenesis, treatment strategies, and hence prognosis. Henceforth, a clear cut and thorough differentiation is absolutely essential. Non-infectious hypopyon, or sterile hypopyon, is not caused by living pathogenic organisms. The causes include things like being after an operation, responses caused by drugs, immune disorders and tumours inside the eye.

#### 3.2.1 Hypopyon following intraocular injection or surgery

Sterile (non-infectious) hypopyon may develop in a subset of patients following intraocular therapeutic interventions, most notably intravitreal administration of anti-vascular endothelial growth factor (anti-VEGF) agents and intraocular silicone oil tamponade. The pathogenesis is generally attributed to either direct irritative effects of the administered agents or immune-mediated inflammatory reactions. Clinically, this form of hypopyon is typically associated with mild symptoms and follows a self-limited clinical course, presenting as a mild anterior chamber reaction with inflammatory cells and flare; microbial cultures of aqueous specimens are uniformly negative. Comparable sterile inflammatory manifestations also occur in the setting of toxic anterior segment syndrome (TASS), a well-recognized postoperative inflammatory entity. Initially described in the 1980s, TASS was first characterized as a sterile hypopyon reaction secondary to residual intraocular polishing compounds (98). It is now defined as an acute, aseptic postoperative inflammatory reaction restricted to the anterior segment, of which hypopyon represents a cardinal clinical feature, accompanied in most cases by corneal edema (99, 100). In a retrospective analysis investigating an outbreak of TASS following vitreoretinal surgery, Kannan et al. documented a strikingly high prevalence of anterior segment inflammatory signs. Among 14 patients with confirmed TASS, hypopyon was present in 13 cases (92.8%), while corneal edema was observed in 78.6% of the cohort, reinforcing hypopyon as a hallmark presenting feature of this sterile postoperative condition (101). Topical corticosteroid eye drops are the mainstay of the clinical management of this condition. This can be supplemented by the anterior chamber washout in cases with more pronounced features. Despite the clinical presentation of an abscess or other local infection, they should not be regarded as a form of infectious endophthalmitis. However, it is essential to maintain a high index of suspicion and distinguish them from infectious endophthalmitis very vigilantly, as a misdiagnosis could result in either the unwarranted use of antibiotic therapy or a dangerous delay to initiate correct treatment (102–104).

#### 3.2.2 Autoimmune diseases

One of the most important and serious entities seen in clinical ophthalmology is Behçet's disease. The classic manifestation involves recurrent oral and genital aphthous ulcers, skin lesions, and uveitis. It can cause serious widespread effects on other organs as well. Thus, the ophthalmologist must carefully differentiate it from other such conditions. The 1979 Frederick H. Verhoeff Lecture emphasized that hypopyon in Behçet's disease is a classical manifestation of anterior segment involvement (105). Severe attacks of anterior uveitis caused by Behçet may lead to a characteristic hypopyon (accumulation of leukocytic exudate in the anterior chamber) (106). The hypopyon found in Behçet's disease has unique properties that help in identifying it from infectious endophthalmitis and masquerade syndromes. Its key attributes are as follows (105, 107): Morphology and Mobility—Non—viscous and mobile—The hypopyon is rather fleeting and moves with the head. For example, placing a patient in a head-down posture for a few minutes will often result in a clear drop of hypopyon. The exudate in infectious endophthalmitis is usually thick and “fixed” as opposed to this (108).

Self limiting characteristics: These episodes spontaneously resolve after a few days. In general, there is no residual tissue damage after resolution (109). In case of repeated attacks, the individual can develop iris atrophy as a sequel (110). Therefore, this finding alone should not justify a strategy of observation, as it often signifies a greater intraocular inflammatory burden and is frequently accompanied by or may herald severe involvement such as posterior or panuveitis and retinal vasculitis. Reliance solely on whether hypopyon resolves rapidly is insufficient to determine overall disease severity and long-term prognosis; systematic evaluation and prompt initiation of appropriate therapy are therefore warranted.

Associated findings: Hypopyon commonly occurs in serous anterior uveitis accompanied by conjunctival hyperemia, sparse inflammatory cells, and keratic precipitates, a clinical phenotype also referred to as “hot hypopyon”. However, active anterior uveitis secondary to Behçet disease cannot be excluded even in the absence of clinically apparent conjunctival hyperemia, as hypopyon in Behçet uveitis may also present as “cold hypopyon”—a distinguishing feature that aids in the differential diagnosis from other forms of anterior uveitis (108). Ocular Behçet disease is typically bilateral and relapsing; even when only one eye is affected at onset, this does not indicate a low-risk profile, as most patients subsequently develop bilateral involvement and should therefore be managed and followed as a potentially bilateral disease, although the reported rates of bilateral involvement vary considerably across cohorts (111, 112).

Although hypopyon is classically associated with infectious endophthalmitis and Behçet's disease, non-infectious aetiologies—most notably HLA-B27-associated acute anterior uveitis (AAU)—account for a substantial proportion of clinical presentations. In a large retrospective cohort study of 426 consecutive hypopyon episodes presenting to an acute ophthalmology emergency department, Pépin et al. identified HLA-B27-associated uveitis as the single most common underlying cause, responsible for 34.3% (146 episodes) of all cases. Infectious keratitis and infectious endophthalmitis followed as the next most frequent aetiologies, accounting for 29.3% and 14.8% of episodes, respectively (113). From a diagnostic standpoint, Caplash and colleagues have highlighted that severe presentations of HLA-B27-associated AAU with dense anterior segment inflammation may masquerade as endogenous endophthalmitis, particularly when extensive fibrin deposition and/or hypopyon impairs fundoscopic visualization (114). This diagnostic challenge is further underscored by epidemiological evidence demonstrating that hypopyon is well documented in severe flares of spondyloarthritis (SpA)-related and HLA-B27-associated anterior uveitis. The 10-year cumulative incidence of hypopyon uveitis in patients with HLA-B27-associated disease has been estimated at 17%, with both SpA phenotype and HLA-B27 positivity confirmed as independent risk factors (115, 116). Overall, HLA-B27-associated anterior uveitis represents the most prevalent subtype of hypopyon uveitis, constituting approximately 37% of all reported cases (115). Around 18% of patients with HLA-B27-associated uveitis will experience at least one episode complicated by hypopyon over their disease course, and HLA-B27 positivity confers an approximately two-fold increased risk of hypopyon development (117, 118). In severe acute attacks of AAU, hypopyon is observed in 14%–18% of affected eyes (119, 120). Taken together, these findings underscore the clinical imperative for accurate differentiation between infectious and non-infectious causes of hypopyon. Proficiency in the diagnosis and management of sterile hypopyon is an indispensable component of clinical practice, as it directly supports optimized patient care and improved long-term visual outcomes.

## 4 Classification by origin: primary and secondary hypopyon

Hypopyon is basically classified into two types: Infective and Non-infective, but further classification depending upon the site and sequence of the disease is of clinical value. It is very crucial to categorise the process as primary or secondary as it helps the clinician identify the causative pathogen and the involved parts primarily. The precision of the diagnosis is crucial as it steers the diagnostic workup, reduces the chances of it being wrong, ensures the right therapy is offered and not one that is ineffective or harmful, allows accurate prognosis, and helps with the follow-up.

### 4.1 Primary hypopyon

A primary hypopyon is one in which the eye is the site of primary involvement. When a pathogen or a disease process that affects the eye gets in, it results in an inflammatory response inside the eye which produces hypopyon. A pathogen could be a micro-organism, or the disease process could be a tumour. As discussed before, many of the examples fall in this category. These are infectious hypopyon, non-infectious hypopyon following intraocular injections or surgery, hypopyon in specific auto-immune disease and hypopyon from intraocular tumors. All these agents share a commonality: the inflammatory insult is generated within the eye, and hypopyon is formed. It is important for the eye doctor to produce a proper differential diagnosis and give etiology directed treatment. Clinical decision-making must be guided by clinical judgment and phenotypic presentation: corticosteroids must not be initiated until infection has been definitively ruled out. Among patients with confirmed infection receiving targeted antimicrobial therapy, adjunctive intravitreal dexamethasone remains controversial due to strong pathogen-dependent differences in efficacy and safety. It is supported as an optional adjunct for moderate-to-severe bacterial endophthalmitis (endorsed by ESCRS guidelines) to accelerate inflammatory resolution and reduce late proliferative complications, but is contraindicated in fungal and oomycete (P. insidiosum) infections due to impaired pathogen containment and worse outcomes. Risks include masking of disease progression and steroid-related adverse events, leading to a consensus stratified approach tailored to etiological confirmation and disease severity. Corticosteroids should be very carefully used to manage non-infectious inflammation (like autoimmune) only after a very strong ruling out of an infectious cause. Additionally, the close monitoring status and follow-up of the patient is essential to make changes in the treatment on-time. As these principles of management have already been discussed, we shall not go into detail about them.

The intraocular tumors will be focused on in this section on primary hypopyon. A classic and critical example is Retinoblastoma. The retina is the source of this tumor which is the most common primary intraocular malignancy of infancy and childhood. The occurrence of two common symptoms should make the physician think of a diagnosis of retinoblastoma. These are: “Leukocoria” or “white eye reflex”; and “Strabismus” or “misalignment of the eyes” (121).

Fundus examination mainly helps in making a diagnosis. Imaging techniques including ultrasound, MRI, and CT, are important supplementary means of confirmation and staging. A thorough fundoscopic examination may be performed under general anesthesia in children to arrive at a definite diagnosis. A white retinal mass characterises the lesion which has vasculature with “fish-flesh” aneurysmal dilatations. The B-scan ultrasonography of the eye shows the tumor appearing as a well-defined mass with relatively higher reflectivity than that of the vitreous body. An important characteristic which is highly suggestive, and often pathognomonic, is the presence of intrinsic fine calcifications within the lesion (121). In this growth pattern, secondary retinal detachment is a common finding. Magnetic Resonance Imaging (MRI) is the best imaging technique to assess the precise local extent of the disease and differential diagnosis. When a tumor is examined with an MRI scan on T1-weighted sequences, it has an iso- to slightly hyperintense signal intensity compared to the vitreous humor of the eye. Conversely, on T2-weighted sequences, it shows a characteristically hypointense signal relative to the vitreous. Visualization of associated retinal detachment may also be possible (121). The imaging profile is needed to differentiate retinoblastoma from other simulating conditions. Furthermore, helps in determining any extraocular extension/optic nerve involvement. An aqueous humor biopsy and genetic tests are needed for diagnosis if the processes mentioned above do not yield the definitive diagnosis (122). In addition to the above screening method, Rb should be distinguished from persistent hyperplastic primary vitreous (PHPV) also to avoid misdiagnosis.

### 4.2 Secondary hypopyon

Hypopyon that occurs as a sequel of intraocular inflammatory disease in which the eye is not primarily involved or infected is known as a secondary hypopyon. Cases arising from tumor invasion from other sites in the body are one category. The second category includes a pathogen that invades the body, arising due to reduction in the immunity like that in CML or HIV infection. Moreover, organisms such as Treponema pallidum or Mycobacterium tuberculosis (that do not infect the eye directly), bacteremia and sepsis can also affect the eye and cause ocular manifestations.

According to research by Dr. Mohamad A. Yassin and the group, ocular involvement is common in chronic myelogenous leukemia (CML) (123). However, few doctors have delved into this topic. It is cited that the intraocular symptoms due to CML (chronic myelogenous leukaemia) must be differentiated from infectious diseases and other non-infectious diseases. The fundamental unique features depend upon clinical manifestations, laboratory examination, and imaging evaluation.

Most of the key clinical features of CML-related hypopyon is associated with the initial presentation of CML or blast crisis phase signs. A condition represented by elevated white blood cell count in the blood is hyperleukocytosis. It is often characterized by anterior uveitis and iris neovascularization. In some cases, “hyphema-hypopyon” (not the usual purulent hypopyon) may be observed (124, 125).

Symptomatic Characteristic: The pain is mild (occurs in only 6% of cases), conjunctival hyperemia is not manifest and there is no obvious ocular surface infection foci (for instance, corneal ulcer). The hypopyon is usually heterogeneous or blood-stained which is atypical of the yellow-white pus. The aqueous humor in the eye has cytology sampling. It has the predominant cell type—neutrophils, with no malignant tumor cells or pathogens, bacteria or fungi. The complete blood count (CBC) shows a significant increase in white blood cells (WBC) with myeloid immature cells. This (BCR-ABL fusion gene) is detected which is the diagnostic basis of CML. The following is a comparison table of diseases commonly requiring differentiation from CML in clinical practice (Table 6).

**Table 6:** Differential diagnosis of ocular symptoms caused by different diseases.

Differential dimensions	Chronic myelogenous leukemia (CML)	Infectious endophthalmitis	Diabetic-related ocular diseases	Non-infectious anterior uveitis
Clinical symptoms	Pain is mild with no obvious eye pain; conjunctival hyperemia is not significant.	Symptoms are severe: 74% of patients present with severe pain, 82% have obvious conjunctival hyperemia; most have a history of worsening ocular discomfort (126–128)	Accompanied by diabetes-related symptoms; ocular pain is mild; associated with poor blood glucose control	History of recurrent episodes, with mild to moderate pain; accompanied by blurred vision; rarely occur acute infection-like symptoms (129)
Ocular signs	Hypopyon: mostly heterogeneous or blood-stained (different from typical purulent hypopyon), with no obvious stratification; Associated manifestations: often accompanied by iris neovascularization (rubeosis) (124, 125)and leukemic retinopathy (e.g., Roth spots, vascular tortuosity) (125)	Hypopyon: most of cases present as typical yellow-white homogeneous purulent effusion, which may show stratification (130); associated manifestations: most cases have an ocular surface infection focus (e.g., corneal ulcer) or a history of ophthalmic surgery/trauma, with obvious vitreous opacity	Hypopyon: small in amount, accompanied by vitreous hemorrhage (131); associated manifestations: presence of proliferative diabetic retinopathy (132)	Hypopyon: rare, mostly presenting as a small amount of inflammatory cell accumulation (forming hypopyon) (133); associated manifestations: may be accompanied by posterior synechia of the iris and lens opacity (134), no CML-related fundus changes
Systemic and laboratory examinations	CBC:WBC count is significantly elevated, accompanied by myeloid immature cells; Bone Marrow Biopsy: BCR-ABL fusion gene is detected;	CBC: Most cases show no WBC abnormalities; severe infection may be accompanied by elevated WBC count; Aqueous Humor Culture: Bacteria/fungi can be detected No BCR-ABL fusion gene is present	CBC: No CML-related abnormalities Blood Glucose: Fasting blood glucose/glycated hemoglobin (HbA1c) is significantly elevated;	CBC: Normal, with no CML-related white blood cell (WBC) abnormalities Inflammatory markers [e.g., C-reactive protein (CRP)] may be slightly elevated BCR-ABL fusion gene
Therapeutic response	Short-term anti-inflammatory; Primary disease control → Hypopyon resolves within weeks	Corticosteroids contraindicated until infection ruled out. Intravitreal antibiotics; hypopyon resolves in 1–2 weeks (if effective)	Topical corticosteroids glucose control + Anti-VEGF/PRP	Effective; symptoms resolve in 1–2 weeks systemic hematological treatment unnecessary

Table 6 summarizes the differential diagnostic features of hypopyon and associated ocular manifestations across four distinct clinical entities—chronic myelogenous leukemia (CML), infectious endophthalmitis, diabetic-related ocular diseases, and non-infectious anterior uveitis—stratified by clinical symptoms, ocular signs, systemic and laboratory findings, and therapeutic response profiles. Clinically, ocular involvement secondary to CML typically presents with mild discomfort, in the absence of prominent pain or pronounced conjunctival hyperemia. Infectious endophthalmitis, by contrast, follows a severe clinical phenotype: severe ocular pain is reported in 74% of cases, marked conjunctival hyperemia in 82%, and hypopyon formation in 86% (126–128).

Diabetic-related ocular diseases are accompanied by systemic diabetic manifestations and mild ocular pain, with episodes closely linked to poor glycemic control, and non-infectious anterior uveitis follows a typically recurrent course with mild to moderate pain and blurred vision, while acute infection-like presentations are rare (129). With respect to ocular signs, the hypopyon associated with CML is typically heterogeneous or blood-stained, lacking the distinct stratification seen in classic purulent hypopyon, and is frequently accompanied by iris neovascularization (rubeosis iridis) and leukemic retinopathy including Roth spots and vascular tortuosity (124, 125). In infectious endophthalmitis, most of cases present with a characteristic yellow-white, homogeneous purulent hypopyon that may exhibit layering (130), along with an identifiable ocular surface infectious focus (e.g., corneal ulcer) or a history of prior ophthalmic surgery or trauma, and marked vitreous opacity is commonly observed. For diabetic-related ocular diseases, hypopyon is typically small in volume and accompanied by vitreous hemorrhage, in the setting of underlying proliferative diabetic retinopathy (131, 132).

In non-infectious anterior uveitis, hypopyon is uncommon and, when present, consists of a small accumulation of inflammatory cells, with associated findings including posterior synechiae and lens opacity but no CML-related fundus abnormalities (133, 134). Systemic and laboratory examinations further distinguish these entities: CML is marked by markedly elevated white blood cell (WBC) counts with myeloid immature cells on complete blood count (CBC) and detection of the BCR-ABL fusion gene on bone marrow biopsy, while infectious endophthalmitis generally shows no WBC abnormalities on CBC (though counts may be elevated in severe infection), yields positive bacterial or fungal growth on aqueous humor culture, and lacks the BCR-ABL fusion gene; diabetic-related ocular diseases show no CML-associated hematologic abnormalities but significantly elevated fasting blood glucose and glycated hemoglobin (HbA1c) (135), and non-infectious anterior uveitis typically presents with a normal CBC, no CML-related WBC alterations, possible mild elevation of inflammatory markers such as C-reactive protein (CRP), and absence of the BCR-ABL fusion gene.

Finally, therapeutic response patterns support differential diagnosis: hypopyon in CML resolves within weeks following short-term anti-inflammatory therapy and adequate control of the underlying hematologic malignancy, whereas empiric anti-inflammatory therapy is contraindicated in infectious endophthalmitis due to the risk of exacerbating infection, and intravitreal antibiotic administration typically leads to hypopyon resolution within 1–2 weeks when effective; diabetic-related ocular diseases respond to topical corticosteroids combined with glycemic control and adjunctive therapies such as anti-VEGF agents or PRP. Non-infectious anterior uveitis shows a favorable response to anti-inflammatory therapy with symptom resolution within 1–2 weeks, with no requirement for systemic hematologic treatment (136). Severe, refractory, or recurrent cases, as well as those complicated by underlying systemic diseases, require systemic anti-inflammatory and immunosuppressive therapy. Juvenile idiopathic arthritis (JIA) is a well-established risk factor for this subtype of anterior uveitis. JIA-associated uveitis (JIA-U) typically has an insidious onset and follows a chronic, relapsing course, necessitating long-term follow-up and management. Guided by a stepwise treatment paradigm, therapy escalates from topical corticosteroids to systemic immunosuppressive agents, and long-term standardized management can reduce the risk of blinding ocular complications (137).

## 5 Classification by pathological nature: true hypopyon vs. pseudo-hypopyon

These are probably the most important distinction we must make pathologically. It goes to the core of a disease process and governs all that we do to treat the patient. The inflammatory nature of the accumulated substance is what makes the difference.

Both hypopyon and pseudo-hypopyon appear as layering of sediments in the dependent portion of the anterior chamber. The terms are based on the inflammatory vs. non-inflammatory origin of the sediments (either local ocular or systemic diseases).

Historically, a key terminological challenge has persisted in this field: although the term pseudohypopyon entered clinical practice centuries ago, no universally agreed definition was adopted across the clinical community for an extended period. This lack of standardized nomenclature has historically made it difficult to ascertain the true pathological nature of anterior chamber deposits and contributed to diagnostic confusion. Importantly, true hypopyon and pseudohypopyon differ substantially in their underlying pathogenesis, treatment strategies, and prognostic outcomes. Failure to recognize the distinction early and initiate appropriate management may ultimately result in permanent sight-threatening visual impairment.

### 5.1 True hypopyon (inflammatory)

The specific and classical clinical use of the unmodified term hypopyon is an inflammatory exudate in the anterior chamber. Its appearance is an important sign of an acute ocular disease changing to a late exudative disease. The clinical picture is often dramatic. The patient usually presents with ciliary flush, severe ocular pain, blurred vision, photophobia and epiphora. A true hypopyon really does indicate severe inflammation inside the eye. A significant observation that can be both diagnostic and therapeutic is that this inflammation will often resolve quickly with corticosteroids. The basic mechanism is the disruption of the blood-aqueous barrier at the level of the capillaries in the iris and ciliary body. The non-pigmented ciliary epithelium, posterior iris epithelium, and iris endothelial cells no longer maintain their integrity, which causes a massive increase in vascular permeability. When the disease is active, the hypopyon is mainly made of inflammatory white blood cells. It refers mostly to polymorphonuclear neutrophils. These cells are recruited from uveal blood vessels. The exudate also consists of tissue debris, proteins, fibrin and a series of inflammatory mediators. Hypopyon typically contains the offending organisms, as well as their metabolites in infections. In the later stages, there may be a change to lymphocytic infiltration.

Clinical Correlations:Sterile hypopyon is classically linked to ocular behçet's disease (OBD) and HLA-B27 associated acute anterior uveitis (AAU).

Infectious Hypopyon most commonly arises from external (for example, surgery and trauma) or internal (blood-borne spread) bacterial infections in the eye. A true ophthalmic emergency, this one carries high risk of vision-threatening complications and permanent visual loss (3, 118).

### 5.2 Pseudohypopyon (non-inflammatory)

On the other hand, pseudo-hypopyon refers to a non-inflammatory sediment in the anterior chamber that is often chronic and mostly painless (138). One of the classic findings in “masquerade syndromes”, particularly those associated with ocular/systemic malignancy. Minimal or lack of response to corticosteroid therapy is a vital clinical clue for diagnosis.

This has been historically referred to as non-infectious hypopyon or tumor-associated hypopyon in the literature. However, due to the fact that the lesion is very likely not inflammatory in nature, “pseudo-hypopyon” is pathologically a more correct term and is to be preferred once confirmed. Although it may look like a sterile inflammatory hypopyon, “non-inflammatory hypopyon” is a misnomer. Some neoplastic pseudo-hypopyons may have an additional inflammatory component caused by damage or degeneration in the uveal tract.

Sediment composition is essentially different. It is primarily made of neoplastic cells from leukemia, lymphoma, or retinoblastoma. True hypopyon consists of inflammatory exudate and may be sterile or associated with infection; pseudohypopyon consists predominantly of non-inflammatory material. This cellular make-up is the basis for its unique clinical behaviour and management. Understanding this distinction is not about semantics, but rather a vital diagnostic tool that changes management from anti-inflammatory to oncological evaluation and treatment (3). The differential diagnosis is as follows: (Table 7) (3).

**Table 7:** Different characteristics of hypopyon and pseudohypopyon.

Feature	Hypopyon	Pseudohypopyon
Component	Inflammatory deposits (containing neutrophils, fibrin, and inflammatory mediators; may contain microorganisms)	Non-inflammatory deposits (containing neoplastic cells, cholesterol crystals, silicone oil, etc.; no microorganisms present)
Common causes	Ocular Behçet's Disease (OBD), HLA-B27-associated Acute Anterior Uveitis (AAU), infectious endophthalmitis (bacterial/fungal), and Toxic Anterior Segment Syndrome (TASS)	Leukemia, vitreoretinal lymphoma, retinoblastoma, cholesterol endophthalmitis, and steroid crystals after intravitreal triamcinolone acetonide (IVTA) injection
Response to hormones	Non-infectious cases (e.g., OBD) are sensitive to hormones, while infectious cases require anti-infective treatment first, followed by anti-inflammatory therapy	No response or only a mild response
Typical characteristics	Mobility or immobility, heat/cold and white/yellow	Rapid mobility, cold type, white/black and massive/wavy
Systemic associations	Autoimmune diseases and infections	Hematological malignancies, and solid tumor metastases

## 6 Classification based on the characteristics of hypopyon

1. Classification by “visibility” (the most basic differential dimension)

There are different types of hypopyon, based on its size (depending on what you can observe using a slit lamp) and location (specific point) (Table 8).

2. Classification by “activity (mobility)” (a core differential dimension)

**Table 8:** The visibility of hypopyon and pseudohypopyon.

Classification	Definition	Clinical features
Macroscopic hypopyon/pseudohypopyon (macro-)	Visible to the naked eye (when in an upright position, it is mostly located at the 6 o'clock position; some can only be seen when looking downward)	The thickness of the deposit is ≥1 mm, which is easy to detect, such as the “white horizontal layered hypopyon” in patients with OBD
Microscopic hypopyon/pseudohypopyon (micro-)	Not visible to the naked eye, only observable under a slit-lamp biomicroscope	The thickness of the deposit is less than 1 mm, presenting as “fine granular”, such as the “trace hypopyon” in the early stage of AAU
Occult hypopyon/pseudohypopyon (occult-)	Not visible under a slit lamp, only observable via gonioscopy	It is most prone to missed diagnosis and requires routine gonioscopy for screening, such as “trace deposits in the anterior chamber angle” in patients with OBD

Adapted from the “naming-meshing” terminological framework for anterior chamber sedimentation proposed by Evereklioglu et al.

Historically, microbial endophthalmitis and non-infectious inflammation lead to “fixed, immobile (plastic) hypopyon”. During the onset of inflammation, the fibrinous hypopyon contains a significant amount of fibrinous exudate. On the other hand, mobile or displaceable hypopyon in the anterior chamber is described as having aqueous flare and composed of serous proteins (Table 9).

3. Classification by “Color” (indicating specific etiologies)

**Table 9:** The mobility of hypopyon and pseudohypopyon.

Classification	Definition	Clinical features
Shifting	After changes in body position or head shaking, the deposits shift or disperse within a few minutes	Characteristic manifestation of OBD: Horizontal layered hypopyon shifts to the lower position after head tilting, and can move to the lateral side of the eye when lying completely on one's side; Pseudohypopyon: Deposits of neoplastic cells disperse within a few seconds after head shaking
Non-shifting	The deposits remain immobile after changes in body position	It has a sticky texture, which is fixed at the 6 o'clock position without displacement

Adapted from the “naming-meshing” terminological framework for anterior chamber sedimentation proposed by Evereklioglu et al.

Hypopyon refers to inflammatory deposits. The colour of the hypopyon is mostly related to the nature of the inflammation (infectious/non-infectious) and the type of pathogen. Pseudohypopyon word refers to the presence of deposits in the anterior chamber that do not elicit an inflammatory reaction. The colour of pseudohypopyon deposits is usually dependent on what is composing them, that is, neoplastic cells, crystals, pigment granules, etc. (Table 10).

4. Classification by “Shape and Location” (highly specific to etiologies)

**Table 10:** The color of hypopyon and pseudohypopyon.

Color	Definition	Clinical features
White/Off-white	It is the most common color	Hypopyon: OBD with “pure white horizontal layered” appearance, AAU with “off-white granular” appearance; Pseudohypopyon: Retinoblastoma with “massive white patch” appearance
Pink	It appears “light pink” when there is no bleeding and “blood-speckled” when accompanied by bleeding	Characteristics of Serratia marcescens infection: The hypopyon contains red pigments produced by bacteria, and still presents as pink even without bleeding; Ocular toxocariasis: Associated with retinal infiltration
Yellow	It contains pus or hemolytic products and presents as “golden yellow or dark yellow”	Characteristics of panophthalmitis: Accompanied by severe conjunctival hyperemia and corneal edema; for example, “yellow suppurative hypopyon” is seen in bacterial panophthalmitis.
Black	It contains melanin granules or necrotic neoplastic cells and presents as “brownish-black to pure black”	Characteristics of uveal melanoma: Accompanied by iris pigment loss, with deposits adhering to the corneal endothelium.

Adapted from the “naming-meshing” terminological framework for anterior chamber sedimentation proposed by Evereklioglu et al.

The deposits' features are determined by their constitution (inflammatory exudate/neoplastic cells/crystals), viscosity, and the environment of the affected local anatomy; the different shapes may be reflective of the directional etiology. Depositional site is directly related to the anatomical structure (cornea, anterior chamber, capsule, posterior segment, etc.) and the site of invasion of the etiology; different sites correspond to different lesion types (Table 11).

5. Classification by “Scleral Manifestations (Hot/Cold)” (for Rapid Differentiation of Inflammatory Properties)

**Table 11:** The shape and location of hypopyon and pseudohypopyon.

Shape and location	Definition	Clinical features
Horizontal layered	When in an upright position, it presents as a “horizontal strip” along the lower part of the anterior chamber, with neat edges or slight elevation	The most classic shape, such as the “pure white horizontal layer” in OBD and the “off-white horizontal layer” in AAU
Pyramidal shape	It is raised in the center with sharp edges, accompanied by “geographic infiltration” and satellite lesions in the cornea	Unique to fungal keratitis: For example, Aspergillus keratitis presents with a “white pyramidal hypopyon,” where the corneal infiltration area is connected to the hypopyon
Annular	It is distributed in an “annular pattern” along the periphery of the anterior chamber, with a transparent center	Rare, characteristics of meningococcal endophthalmitis: accompanied by systemic infection symptoms (fever, rash)
Inverted	The deposit is located in the upper part of the anterior chamber, as the density of the deposit is lower than that of the aqueous humor	Characteristics of silicone oil emulsification: After vitrectomy, silicone oil enters the anterior chamber, presenting as “white punctate inverted deposits”;Infectious scleritis: Accompanied by scleral hyperemia

Adapted from the “naming-meshing” terminological framework for anterior chamber sedimentation proposed by Evereklioglu et al.

Hot hypopyon is characterized by prominent ocular inflammation, typically accompanied by conjunctival or ciliary hyperemia. The imprints of inflammation in ocular tissues and structures are in line with ocular surface vascular dilation (hyperemia), in keeping with standard clinical rationale “inflammation → hyperemia”. In most cases of inflammatory hypopyon, this is the expected appearance. Cold type: Refers to hypopyon with no ocular surface hyperemia (normal appearance, presenting as “white eye”). To put it another way, the hypopyon denotes significant intraocular inflammation but the absence of conjunctival hyperaemia means that the ocular surface displays vascular dilation that contradicts the expected response “severe inflammation → calm appearance”. This is a sign of hypopyon due to certain special etiologies. The prominent disease activity can often be easily overlooked due to the “normal appearance”. Both warm and cold hypopyon presentations may occur in Behçet disease (108) (Table 12).

**Table 12:** Changes in hypopyon influenced by the rate of disease progression.

Classification	Definition	Clinical features
Warm	Accompanied by ciliary hyperemia/conjunctival vascular dilation, which suggests acute inflammation or tumor irritation	Such as AAU presenting with “red eye + white hypopyon” and leukemia presenting with “red eye + pink pseudohypopyon”
Cold	No hyperemia (“white eye”), yet there may be severe intraocular inflammation or tumors	Characteristic manifestations of OBD: “white eye + white mobile hypopyon”, which is easily misjudged as “no inflammation”; Neoplastic pseudohypopyon: “white eye + black massive deposits”

Adapted from the “naming-meshing” terminological framework for anterior chamber sedimentation proposed by Evereklioglu et al.

## 7 Limitations

Several methodological limitations of this narrative review should be acknowledged when interpreting the findings and recommendations presented herein.

First, the literature search was restricted exclusively to the PubMed database. No additional databases (including Embase, Cochrane Library) were searched, and grey literature, conference abstracts, and non-English language studies were not systematically considered. This single-database search strategy may have resulted in incomplete coverage of the available evidence and introduced a degree of publication bias.

Second, no formal evidence grading system was applied to rate the quality of included evidence or the strength of clinical recommendations. The included studies varied substantially in study design, sample size, and methodological rigor, and the level of evidence supporting individual conclusions was not systematically stratified. Accordingly, the clinical implications of this review should be interpreted with caution.

Third, the four-dimensional classification framework for hypopyon proposed in this work represents a conceptual synthesis derived from the integration of existing pathological and clinical evidence. This framework has not been formally validated in prospective multicenter clinical studies, and its diagnostic accuracy, inter-observer reliability, and performance in guiding clinical decision-making remain to be established.

Fourth, literature screening, data extraction, and qualitative synthesis were performed by a single research team, without independent dual screening or adjudication of discrepancies. This introduces a potential risk of selection bias and subjective interpretation of the included evidence.

Despite these limitations, this review provides a structured synthesis of the currently available evidence on the pathogenesis, classification, and differential diagnosis of hypopyon, and offers a clinically useful framework to support bedside decision-making. These methodological gaps also highlight priorities for future, more methodologically rigorous investigations in this field.

## 8 Summary and outlook

### 8.1 Summary

Hypopyon has been an important ophthalmic sign from antiquity down to the present and has undergone a change of significance in clinical practice. From a definition of “pus in the anterior chamber”, it has become a diagnostic window which contains abundant etiological and pathological information. The article makes systematically review and synthesis of research progress on hypopyon, with the main findings summarized.

#### 8.1.1 Multi-dimensional and multi-disciplinary joint analysis

This article breaks the shackles of the traditional single classification model and proposes an integrated multi-dimensional classification framework. The system looks to diagnose the cause of the hypopyon, its origin, the nature of the hypopyon and information on the hypopyon like colour, mobility, shape etc. It helps the clinician make the jump from “hypopyon seen” to “hypopyon diagnosed”.

#### 8.1.2 In-depth analysis of pathogenesis

The article explains how hypopyon is caused by pathogens (fungi, bacteria, saprophytes, viruses) and by non-infectious causes (autoimmune disease, tumour, drug reaction). It places a particular emphasis on how the virulence factors from different microorganisms (for example, fungal biofilms, bacterial pigments, viral cell tropism) interact with the host immune responses (for example, acute storms dominated by neutrophils, chronic granulomas dominated by macrophages), which determines hypopyon features and disease prognosis.

#### 8.1.3 Refined evolution of diagnostic strategies

The application of current diagnostic technology, which employs molecular testing for intraocular fluid (PCR), *in vivo* confocal microscopy (IVCM), and high-resolution imaging (AS-OCT), is necessary for etiological diagnosis. On the other hand, it offers naming-meshing system proposed by Evereklioglu et al. This system is a direct linking of objective characteristics of hypopyon e.g., “mobile white layered” for Behçet's disease, “fixed yellow-green” for Pseudomonas aeruginosa infection, to possible etiologies which greatly promotes early and accurate decision-making in diagnosis.

In conclusion, there has been a systemic refinement and mechanism-based understanding of hypopyon. A clinical diagnosis and treatment should be based on multi-dimensional classification, harness advanced technology, and be derived from a deep understanding of underlying pathophysiological process. Clinicians should formulate the most targeted individualized treatment plans.

### 8.2 Outlook

Significant progress in hypopyon knowledge has been made; however, many areas remain and one will face “challenges and opportunities” in the future.

#### 8.2.1 Different schools of thought or controversies

Two core contradictory academic viewpoints exist on hypopyon diagnosis and classification. One camp prioritizes etiological confirmation as the only diagnostic foundation, while the other advocates phenotype-based rapid bedside stratification via hypopyon mobility, color and shape. There is also ongoing debate on the definition boundary of pseudohypopyon, and disagreement over the timing and dosage of adjuvant corticosteroids for infectious hypopyon, lacking unified high-level clinical evidence to reach consensus. In addition, researchers hold divided opinions on whether molecular PCR or microbial culture should serve as the primary diagnostic gold standard for intraocular pathogens.

#### 8.2.2 Fundamental concepts, issues, and problems

The field suffers from disordered, non-uniform terminology of hypopyon, sterile hypopyon and pseudohypopyon, hindering cross-study comparison. The molecular and biophysical mechanisms linking pathogen traits to hypopyon viscosity, color and mobility remain unclear. No unified grading criteria for hypopyon volume and mobility testing have been established. Clinicians also face persistent unsolved dilemmas, including difficult differentiation of culture-negative infection and sterile inflammation, as well as atypical hypopyon manifestations in immunocompromised hosts.

#### 8.2.3 Current research gaps

Mechanistic studies on emerging oomycete pathogens such as Pythium insidiosum are insufficient, with few systematic cohort data describing its hypopyon characteristics. Validated rapid aqueous biomarkers for pathogen differentiation are absent. Existing multi-dimensional classification systems lack large-sample multicenter verification. Uniform intervention thresholds for anterior chamber lavage remain undefined, and hypopyon features in children and immunosuppressed populations are insufficiently characterized. Quantitative imaging indicators and AI auxiliary diagnosis of hypopyon are still in preliminary exploration.

#### 8.2.4 Potential developments in the field

Future research will focus on multiplex microfluidic chips to realize ultra-fast simultaneous screening of bacteria, fungi, viruses and oomycetes. Global multi-center trials will validate phenotype classification systems and formulate unified international terminology and grading consensus. Targeted anti-virulence and anti-inflammatory agents will be developed to reduce hypopyon formation and related complications. Multimodal artificial intelligence models combining slit-lamp and AS-OCT images will achieve automatic etiology prediction. Multidisciplinary collaborative diagnosis workflows will be popularized for recurrent and masquerade hypopyon to optimize individualized visual protection strategies.

In the future, the competent treatment of hypopyon will inevitably be more efficient with the rapid development of multi-omics, molecular imaging and intelligent diagnostic technologies. Through the close integration of macroscopic clinical signs and microscopic molecular mechanisms, we expect to achieve the ideal state of “seeing its form, understanding its cause, and putting in targeted treatment”, thus saving the patient's visual function to the maximum extent.
